# Defective Interfering Genomes and the Full-Length Viral Genome Trigger RIG-I After Infection With Vesicular Stomatitis Virus in a Replication Dependent Manner

**DOI:** 10.3389/fimmu.2021.595390

**Published:** 2021-04-30

**Authors:** Andreas Linder, Viktoria Bothe, Nicolas Linder, Paul Schwarzlmueller, Frank Dahlström, Christoph Bartenhagen, Martin Dugas, Dharmendra Pandey, Julia Thorn-Seshold, Daniel F. R. Boehmer, Lars M. Koenig, Sebastian Kobold, Max Schnurr, Johannes Raedler, Giulia Spielmann, Hadi Karimzadeh, Andreas Schmidt, Stefan Endres, Simon Rothenfusser

**Affiliations:** ^1^ Division of Clinical Pharmacology, University Hospital, LMU Munich, Munich, Germany; ^2^ Department of Medicine II, University Hospital, LMU Munich, Munich, Germany; ^3^ Institute of Medical Informatics, University of Münster, Münster, Germany; ^4^ Einheit für Klinische Pharmakologie (EKLiP), Helmholtz Zentrum München, German Research Center for Environmental Health (HMGU), Neuherberg, Germany; ^5^ German Center for Translational Cancer Research (DKTK), Partner Site Munich, Munich, Germany

**Keywords:** vesicular stomatitis virus (VSV), retinoid acid inducible gene I (RIG-I), pattern recognition receptor (PRR), pathogen associated molecular pattern (PAMP), nucleic acid sensing, defective interfering genome

## Abstract

Replication competent vesicular stomatitis virus (VSV) is the basis of a vaccine against Ebola and VSV strains are developed as oncolytic viruses. Both functions depend on the ability of VSV to induce adequate amounts of interferon-α/β. It is therefore important to understand how VSV triggers interferon responses. VSV activates innate immunity *via* retinoic acid-inducible gene I (RIG-I), a sensor for viral RNA. Our results show that VSV needs to replicate for a robust interferon response. Analysis of RIG-I-associated RNA identified a copy-back defective-interfering (DI) genome and full-length viral genomes as main trigger of RIG-I. VSV stocks depleted of DI genomes lost most of their interferon-stimulating activity. The remaining full-length genome and leader-N-read-through sequences, however, still triggered RIG-I. Awareness for DI genomes as trigger of innate immune responses will help to standardize DI genome content and to purposefully deplete or use DI genomes as natural adjuvants in VSV-based therapeutics.

**Graphical Abstract:**
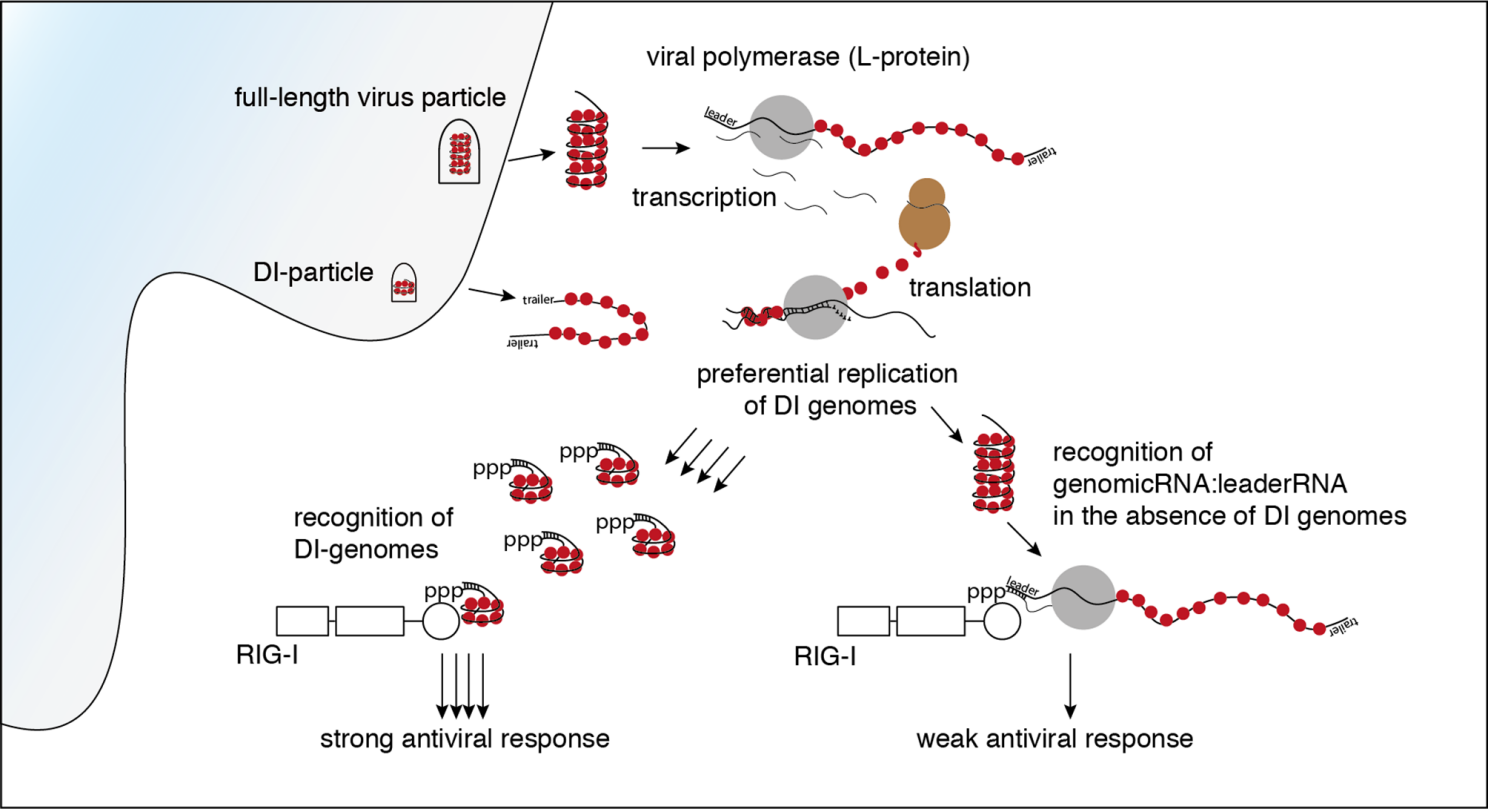


## Introduction

Vesicular stomatitis virus (VSV) is a non-segmented negative-stranded RNA virus that belongs to the family rhabdoviridae (1). It is characterized by a simple structure, the ability to tolerate additional transcriptional units, rapid replication kinetics in mammalian and many other cell types and no relevant clinical pathology in humans. These properties make VSV a frequently used tool for molecular and cellular biologists and an intensely studied target for drug development. VSV is now under development as a backbone vector for live vaccines and proved its great potential in the recent outbreaks of Ebola virus disease. The rVSVΔG-ZEBOV-GP vaccine, in which the VSV envelope glycoprotein is replaced by the Zaire Ebola virus (ZEBOV) glycoprotein (GP) was shown to be highly protective in trials performed in Guinea in 2015 (2), and got approval by the FDA and EMA at the end of 2019. The sustained Ebola GP-specific antibody response induced by the vaccine thereby correlates with an early activation of innate immunity, especially of type-I interferon induced genes (3, 4). This highlights that VSV-based replication-competent vaccines do not only provide a delivery system for the antigen of interest, but serve as an intrinsic adjuvant by stimulating innate immunity. Driven by the success against Ebola several companies and academic groups now work on VSV-based COVID-19 vaccines for the ongoing SARS-CoV-2 pandemic.

In addition, due to an inherent tumor specificity and potential to break immune tolerance in the tumor microenvironment VSV is developed as an oncolytic virus for cancer therapy (5, 6). Oncoselectivity of VSV based on the lower type I IFN-associated antiviral potential of cancer cells and the breakage of tolerance are tightly linked to the ability of VSV to trigger innate immune responses. It is therefore of great interest to understand in detail how VSV is sensed by innate immunity.

Retinoic acid-inducible gene I (RIG-I) is a key cytoplasmic pattern-recognition receptor. Once activated it triggers a signaling cascade that culminates in an antiviral gene program characterized by a type I interferon signature. RIG-I functions as a major sensor for RNA viruses of negative polarity such as influenza A, paramyxoviruses or rhabdoviruses (7, 8) and was shown early on to trigger immune responses upon infection with VSV (9, 10).

As defined by us and others the minimal structural requirements of synthetically produced RIG-I ligands are RNA molecules carrying a 5’-tri- or diphosphate moiety with a blunt-ended double-stranded base pairing of > 20 bp in direct vicinity (11–15).

A wide range of candidate RNAs has since been published to activate RIG-I in infections with Sendai, influenza and measles viruses (13, 16–18). However, so far, the exact nature of the viral RNA-species triggering RIG-I during VSV infection is unknown.

In this study we combine different methods, including deep sequencing, enzyme digestion and length fractionation to investigate the RNA ligands co-immunoprecipitating with RIG-I in VSV-infected cells. This allowed us to identify the RNA-species of VSV that are relevant to activate RIG-I and trigger the interferon responses necessary for its function as live vaccine and oncolytic agent.

## Materials and Methods

### Cell Lines

1205Lu cells were provided by Dr. R. Besch (LMU Munich) and were cultured in DMEM medium supplemented with FCS (10% v/v), L-glutamine (1.5 mM) and ciprofloxacin (10 µg/ml). Flp-In™-T-REx™-293 cells were purchased from Invitrogen and cultured in DMEM medium supplemented as above with additional added zeocin (100 µg/ml) and blasticidin (15 µg/ml). For the generation of HEK 293 FLAG-RIG-I cells, N-terminally FLAG-tagged RIG-I was cloned into the pcDNA™5/FRT/TO vector and Flp-In™-T-REx™-293 cells were cotransfected with this vector along with the pOG44 plasmid. FLAG-RIG-I cells were then cultured in DMEM containing hygromycin (100 µg/ml) and blasticidin. Expression of FLAG-RIG-I was started by the addition of 1 µg/ml tetracycline to the culture medium 24 hours prior to infection. All cell lines were regularly tested for mycoplasma contamination *via* PCR or MycoAlert Mycoplasma Detection Kit (Lonza). Authentication of human cell lines by STR DNA profiling analysis was conducted in house.

1205Lu RIG-I, MDA5 and MAVS knockout cells were generated as described previously (19) by transient transfection of the pCMV-mCherry-Cas9 plasmid (kindly provided by Veit Hornung, Gene Center, LMU Munich), harboring the respective single-guide RNA (sgRNA). PKR-knockout cell lines were produced by transient expression of the enhanced SpCas9(1.1) together with a sgRNA targeting an early exon from T2A-PuroR or -BlastR-modified eSpCas9(1.1) plasmids (Addgene plasmid # 62988 and # 71814, respectively both provided by Feng Zhang). After enrichment by Puromycin/Blasticidin selection for 48 hours, single-cell clones were established by limiting-dilution cultivation and PKR knockouts were confirmed by next-generation sequencing (Illumina MiSeq) of the target locus as described in (20) and *via* western blot. The following sgRNAs were used: DDX58 (RIG-I) GGGTCTTCCGGATATAATCC; IFIH1 (MDA5) GAGGGCTGCCGGTTCTCCGG; MAVS GTACTTCATTGCGGCACTGA; EIF2AK2 (PKR) GTACTACTCCCTGCTTCTGA. Genotypes of the respective knockout cell lines are available upon request.

### Virus Propagation

Vesicular stomatitis Indiana virus (VSV wt) was a gift from A. Krug (LMU Munich), who had received it from D. Kolakofsky`s lab, University of Geneva, Geneva, Switzerland and is originally derived from the Mudd-Summers isolate obtained from cattle in Indiana in 1925. The VSV M51R virus containing a methionine to arginine substitution at positions 51 was a kind gift of Oliver Ebert (TU, Munich). It is derived from the recombinant Indiana strain rVSV generated by Jack Rose (21) and contains a GFP reporter inserted between the G and L gene (22–25). For virus propagation, BHK-21 cells were cultured in GMEM medium supplemented with FCS (10% v/v), tryptose (1%) and penicillin/streptomycin (1%) to a confluency of 80 to 90%. Cells were then infected in Opti-Mem medium at an MOI between 10^-1^ – 10^-4^ PFU/cell. Adsorption of viral particles was allowed to occur for one hour at 37°C temperature before the medium was removed, cells were washed in PBS and GMEM medium was added to the cells. 48-72 hours post infection when infected cells had fully detached virus containing supernatant was collected, centrifuged and stored at -80 C. Virus concentration in stock solutions was determined by standard plaque assay. Both virus strains used as our starting stocks for this study had been propagated for an unknown number of passages with the described protocol unaware of their DI-content without purposefully enriching or depleting DIs.

For the generation of viral stocks with decreased amounts of defective-interfering particles, BHK cells were infected with VSV at an MOI of 10^-6^ PFU/cell. The virus containing supernatant was collected 24 hours post infection. The titer of the resulting viral stock was determined by plaque assay and used for another round of virus production on BHK cells with an MOI of 10^-6^ PFU/cell. This procedure was repeated five times for the wt stock depicted in **Figure 6** and three times for the VSV wt stock and VSV M51R stock depicted in **Supplementary Figure 7**.

### Infection Assays and Cycloheximide Treatment

For infection assays the previous medium was removed and the indicated cell lines were infected with VSV wt or the VSV M51R mutant at an MOI of 1 in Opti-Mem. At the indicated time points, cell supernatant was harvested and cells were lysed to isolate RNA and/or proteins. For immunoprecipitation experiments cells were lysed either 9 or 24 hpi. Where indicated, cycloheximide (100 µg/ml) was added to the medium 30 min prior to infection.

### RNA Extraction

RNA was isolated from cell lysates or eluates from immunoprecipitations using the miRNeasy Mini Kit (QIAGEN) according to the manufacturer’s instructions.

### Retransfection of RNA From VSV-Infected Cells and IP-10 ELISA

To determine the immunostimulatory capacity of VSV-derived RNAs, purified RNA from infected cells was retransfected into 1205Lu cells. For that 1x10^4^ cells plated in a 96-well were either transfected with 100 ng of total cellular RNA or 10 μl of the fragmented RNA (see below). The RNA was diluted in Opti-MEM in a total volume of 20 μl and mixed with 0.3 μl Lipofectamine RNAimax diluted in 20 μl Opti-MEM. After 10 min at room temperature the transfection mix was added to the cells. IP-10 was measured in the supernatant after 24 hours using the OPtEIA IP-10 ELISA kit (BD Biosciences) according to the manufacturer’s instructions.

100 ng/ml high molecular weight polyinosine-polycytidylic acid (poly I:C) from InvivoGen (Toulouse, France) and 500 ng/ml triphosphate RNA (pppRNA) transfected with lipofectamin RNAimax were used as known ligands for MDA5 and RIG-I respectively. pppRNA was synthesized by *in vitro* transcription (IVT) of a double-stranded DNA template (sense: 5’-TAATACGACTCACTATAGCGCTATCCAGCTTACGTAGACTCTACGTAAGCTGGATAGCGC-3’; antisense: 5’-GCGCTATCCAGCTTACGTAGAGCTCTACGTAAGCTGGATAGCGCTATAGTGAGTCGTATTA-3’) as previously described (26).

### Nucleic Acid Modifying Enzymes

For some experiments purified RNA from VSV-infected cells was treated with RNase prior to retransfection into 1205Lu cells. For that 4 µl of RNA in 20 µl buffer was treated for 30 minutes at 37°C with 20 U of RNase R (final concentration: 1 U/µl), 1 U of RNase III (final concentration: 0.05 U/µl) or 1 U of 5’-polyphosphatase (final concentration: 0.05 U/µl) using their respective buffers (Epicentre). RNase III buffer contained 330 mM Tris acetate (pH 7.5), 660 mM potassium acetate, 200 mM magnesium acetate, 5 mM DTT. Buffers for RNase R and 5’-polyphosphatase were provided by the manufacturer along with the enzymes in a 10x stock solution.

### Size-Dependent RNA Isolation and Retransfection

RNA of infected HEK 293 cells (4 x10^6^ cells grown in a 10 cm² dish) or RNA co-immunoprecipitating with RIG-I (see below) was isolated 24 hpi. 8 µg of total RNA diluted in 1.5 x RNA loading dye (98% formamide, 0.025% bromophenol blue, 50 mM EDTA) was incubated at 70°C for 10 min and immediately put on ice. In the case of RNA co-immunoprecipitating with RIG-I all the RNA recovered after immunoprecipitation was used. After 10 min the RNA was separated on a 0.8% agarose gel at 60 V for 2.5 h. Guided by a 10 kbp RNA ladder loaded on the same gel, the RNA was cut size-dependently into ten fractions. RNA was recovered using the *Zymoclean™* gel RNA recovery kit (Zymo Research). Equal volumes of recovered RNA of each slice were retransfected into 1205Lu cells and the immunostimulatory effect was measured as described above.

### Immunoprecipitation of FLAG-RIG-I With Its Associated RNAs

Immunoprecipitation of RIG-I/RNA complexes was performed as described in detail previously (27). Briefly, cells were lysed in lysis buffer (20 mM Tris HCl (pH 7.5), 150 mM NaCl, 0.25% NP-40, 1.5 mM MgCl_2_, 1 mM NaF, 400 U/ml RiboLock RNase Inhibitor (Thermo Scientific), 1:100 Protease Inhibitor Cocktail (SIGMA)). Lysates were precleared with sepharose beads (SIGMA) and incubated at 4°C for two hours with either anti-FLAG M2 Affinity Gel (SIGMA) or IgG control beads (SIGMA). Beads were washed five times in lysis buffer and FLAG-RIG-I was eluted using 3x FLAG peptide (100 µg/ml in Tris-buffered saline [TBS]; 50 mM Tris-Cl, pH 7.6; 150 mM NaCl).

### Immunoblotting

Cellular extracts or eluates from immunoprecipitations were diluted in 2x Laemmli sample buffer (BioRad), separated by SDS-PAGE in 10% Tris-glycine-gels and blotted onto a PVDF-membrane (pore size 0.2 µM, BioRad). After blocking in 5% milk in TBS with 0.1%Tween 20 or 5% BSA (when anti-phospho-IRF3 antibody was used consecutively) for 1 hour at RT. Membranes were then incubated over night at 4°C with the indicated antibodies. After incubation with the corresponding secondary antibody for 1 hour, blots were washed and chemiluminescence was detected with the LAS imager (Fujifilm). Antibodies used in this study were anti-phospho-IRF3 antibody (1:1000, Cell Signalling Technology, CST), anti-VSV G antibody (1:1000, Kerafast), anti-FLAG M2 antibody (1:1000 SIGMA), anti-actin antibody, HRP-linked (1:2000, Santa Cruz), anti-mouse immunoglobulin, HRP-linked (CST), anti-rabbit immunoglobulin, HRP-linked (CST).

### Reverse Transcription and Quantitative PCR

RNA isolated from cell lysates or eluates from immunoprecipitations was reverse-transcribed into cDNA using random hexamer primers (ThermoScientific). As the 51 nucleotides of the leader sequence were too short for the design of a conventional qPCR assay, an additional, partially self-complementary primer, that elongates the cDNA of the leader-RNA by 29 nucleotides, was added to the cDNA synthesis reaction (Leader-RT-primer: GCCTCTCA TGCTGACGAA TTT TGAGAGGC AAAGTTTC; self-complementary regions are underlined). Quantitative PCR assays were designed by the use of the universal probe library design center (UPL, Roche). Primer sequences are detailed in **Supplementary Table 1**. In case of the trailer sequence of VSV no appropriate probe was available in the UPL. Therefore, a custom-made probe was purchased from TIB MOLBIOL. For all qPCR assays, standard curves were generated by the use of serial dilutions of cDNA of VSV-infected HEK 293 cells and deposited in the Lightcycler software. Copy numbers were calculated using the advanced relative quantification algorithm normalized to HPRT using the deposited standard curves. For relative enrichment copies/HPRT values were normalized to the negative control.

### Single Primer cDNA Synthesis and PCR for the Identification of Trailer-Terminated Copy-Back DI-Genomes

To identify trailer-containing copy-back defective interfering genomes in VSV infected cells in an unbiased manner, RNA from cells infected with either VSV wt or VSV M51R was reverse-transcribed with a primer (AAAAAATAAAAACCACAAGAGGGTCTTAAG) that binds RNA-sequences containing the trailer sequence on their 5’-end, i.e. trailer-terminated defective interfering particles and the full length antigenome. In a second step the produced cDNA was PCR-amplified with the same primer leading to the amplification of cDNAs containing the trailer sequence on the 5’-end and its reverse complementary sequence on the 3’-end. PCR products were gel purified and subjected to Sanger sequencing using primers binding at different sites within the L gene matching the region with the expected DI breakpoint based on the size of the observed PCR product (sequencing primer L1: TGAGTAGATTTTCTATACGCCAAGTCTCC; sequencing primer L2: CCAAATACTGCACTCATCTCCTTCAG; sequencing primer L3: ACCCTGTAATTCTACAACGTTTCTCG). The identified breakpoint at genomic position 8103 in VSV M51R stocks was used to design an RT-qPCR assay that allowed for specific quantification of the DI 8103. DI 6494, identified in next generation sequencing experiments, served as a positive control for the assay.

### Preparation of cDNA Libraries and Deep Sequencing

RNA isolated from cell lysates or from FLAG-RIG-I-IPs was processed by removing rRNA with the Ribo-Zero rRNA Removal Kit (Illumina). cDNA libraries were then generated using either the TruSeq Stranded Total RNA Sample Prep Kit (Illumina) or ScriptSeq™ v2 RNA-Seq Library Preparation Kit (Epicentre). Quality of cDNA libraries was assessed on a DNA1500 chip (Bioanalyzer 2100, Agilent). Sequencing was performed by the Laboratory for Functional Genome Analysis (LAFUGA) at the Gene Center Munich on the Illumina Genome Analyzer.

### Alignment and Analysis of Next Generation Sequencing Data

Sequences recovered from the Illumina Genome Analyzer were aligned in two steps with BWA (version 0.7.4) using default parameters. First, a single-end alignment against the human genome (GRCh37.59) identified high-quality nonviral reads. The remaining, unmapped sequences were aligned with BWA-MEM against the VSV reference genome. Coverage was plotted against the genome for every nucleotide or with 50 nucleotid windows or with windows representing the different genes of the VSV genome and the non-coding 3’-leader and 5’-trailer region. Relative enrichment was calculated by dividing coverage from the experimental condition to the control condition for every nucleotide of the VSV genome. Annotations and VSV genome reference were taken from http://www.ncbi.nlm.nih.gov/nuccore/NC_001560.1

RNA-Seq data was deposited in the European Nucleotide Archive and can be accessed *via* the accession number PRJEB42788.

### DI-Genome Breakpoint Detection

For a systematic analysis of breakpoint reads, reads previously unmapped to the human genome were realigned to the VSV genome with the BWA-SW algorithm. It allows the detection of reads, which split up into two sub-sequences that map to two distant locations on the reference genome (split-reads). The distance between the sub-sequences gives information about deleted segments and a different orientation reveals potential inversions. Split-reads supporting the same aberration and break-points were clustered and reported if their BWA-SW mapping quality was greater than 10 (phred-scaled) and the cluster size exceeded 2 reads. The post processing of the split-reads following BWA-SW alignment was done with R.

## Results

### The Kinetics of Immunostimulatory RNA Production and Dependence on Protein Translation Suggest Virus Replication as the Origin of RIG-I Ligands During VSV Infection

It has been shown before that RIG-I is triggered after infection with VSV (9, 10). Using CRISPR-Cas9 edited loss of function variants of the human melanoma cell line 1205Lu, we confirmed that in the experimental system used in this study, the early induction of interferon-stimulated cytokines by VSV is RIG-I- and MAVS-dependent but does not require MDA5 or PKR (see **Supplementary Figure S1**). Several virus-derived RNA species are putative ligands for RIG-I in the replication cycle of VSV. The incoming genome of VSV as the first candidate comprises 11 161 nucleotides. It is tightly wrapped into the ribonucleoprotein (RNP) and encodes five viral proteins: the nucleoprotein (N protein), the phosphoprotein (P protein), the matrix-protein (M protein), the glycoprotein (G protein) and the viral polymerase, also called large protein (L protein). Starting the transcription at the 3’ genomic promoter (leader), the polymerase first gives rise to the 50 nucleotide-long tri-phosphorylated leader RNA and subsequently the monocistronic mRNAs of the five genes. When sufficient N protein has been translated, the polymerase switches from transcription to replication and starts to synthesize the full-length antigenome. At the 3’-end of the antigenome the trailer region serves as promoter for the synthesis of the negative-strand genome (28). Besides these canonical RNAs indispensable for an intact replication cycle, the L protein produces several less well characterized byproducts. These encompass polycistronic mRNAs, naked or encapsidated leader-N read-through products of varying length, a short trailer RNA - an analogue to the leader RNA transcribed from the 3’-end of the antigenome - as well as abortive replication products, so-called defective interfering (DI) RNAs (28).

**Figure 1:**
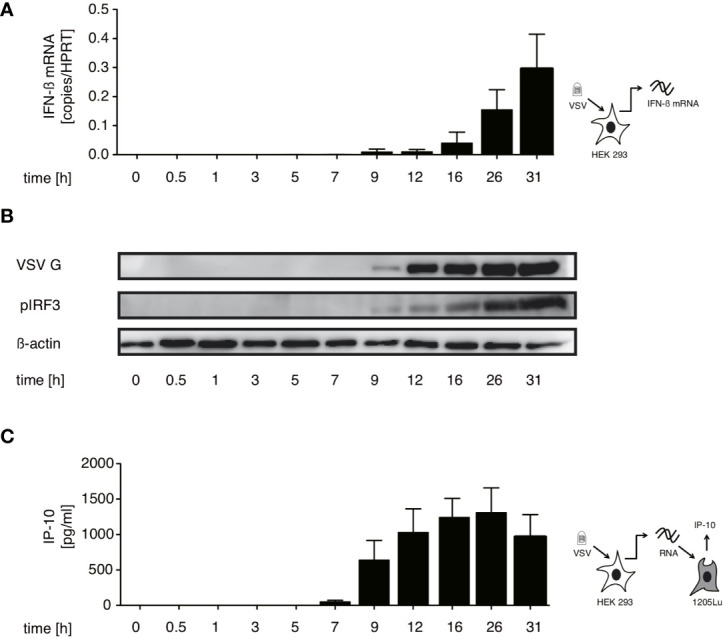
Immunostimulatory RNA production can be detected 7 hours after VSV infection and parallels both IFN-β induction and translation of viral proteins in infected cells. HEK 293 cells were infected with VSV (MOI=1). At the indicated time points post infection cells were lysed, RNA isolated and cytoplasmic and nuclear protein fractions were prepared. **(A)** RNA from infected cells was analyzed by RT-qPCR for the expression of IFN-β. **(B)** Expression of VSV G, IRF3 and β-actin in the cytoplasm and phosphorylated IRF3 in the nuclear fraction was determined by western blotting. **(C)** RNA isolated from VSV-infected HEK 293 cells at the indicated time points after infection was retransfected (100 ng/100 µl) into 1205Lu melanoma cells (8 x 10^3^ cells/well) using RNAiMax Lipofectamin. IP-10 was measured in the supernatant after 24 hours. Data are shown in **(A, C)** as mean +- SD of triplicates and are representative for n=2 independent experiments.

In order to identify which RNA species trigger RIG-I in the course of VSV infection we first searched for the best time point to immunoprecipitate immunostimulatory RNA bound to RIG-I. We therefore performed a time course experiment infecting HEK 293 cells with our stocks of the VSV Indiana strain. Using the production of IFN-β mRNA and phosphorylation of IRF-3 in infected cells as markers for an activated RIG-I signal we detected a first increase in IFN-β transcription 5 hours post infection (hpi) - visible only on an x-axis with logarithmic scale (data not shown). RIG-I-signaling was robustly turned on 9 hpi and increased further till 31 hpi (**Figures 1A, B**).

In addition, isolated cellular RNA of infected cells from the same experiments containing the assumed viral RIG-I ligand was retransfected into 1205Lu melanoma cells to asses when the presence of transferable immunostimulatory RNA could be detected. 1205Lu cells express an intact RIG-I signaling pathway and respond to RIG-I ligands with interferon and IP-10 production (29). The earliest time point at which immunostimulatory RNA could be re-isolated in this system was 7 hpi reaching a peak at around 26 hpi (see **Figure 1C**). The appearance of immunstimulatory RNA thereby coincided with the synthesis of viral proteins measured by western blot of the VSV G protein (see **Figure 1B**).

In the replication cycle of VSV transcription starts almost immediately after infection while replication begins after a lag period of several hours post infection (30–32). The incoming genome or transcription products are available in the cytoplasm very early after the virus enters the cell. The IFN production and occurrence of immunostimulatory RNA after a delay of 5-7 hpi suggests that the RIG-I ligand is likely to be produced during the replication phase of the VSV infection cycle.

**Figure 2:**
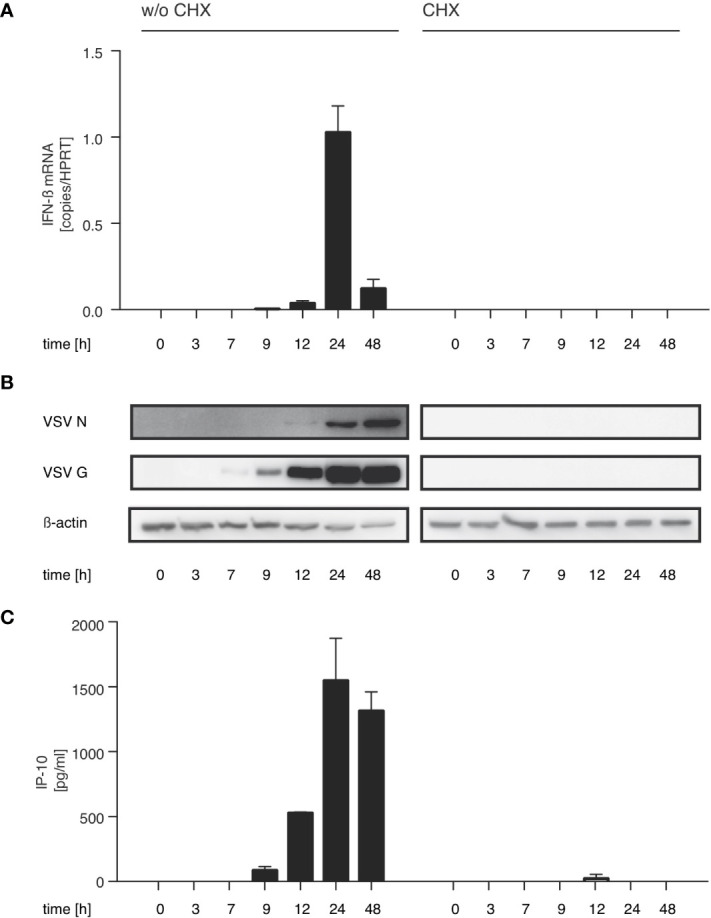
The production of immunostimulatory RNA after VSV infection can be blocked with the translation inhibitor cycloheximide. HEK 293 cells were treated or not with cycloheximide (CHX, 100 µg/ml) 30 min prior to infection with VSV (MOI=1) and lysed at the indicated time points post infection. **(A)** RNA from the infected HEK 293 cells was isolated and analyzed by RT-qPCR for the expression of IFN-β and the reference gene HPRT. **(B)** Protein lysates were analyzed by SDS-PAGE and expression of VSV G, VSV N and β-actin in infected HEK 293 cells was determined by western blotting. **(C)** RNA isolated from VSV-infected HEK 293 cells at the indicated time points after infection was retransfected (100 ng/100 µl) into 1205Lu melanoma cells (8 x 10^3^ cells/well) using RNAiMax Lipofectamin. IP-10 was measured in the supernatant after 24 hours. Data are shown as mean +- SD for n=2 independent experiments.

With this in mind we investigated if the synthesis of immunostimulatory RNAs could be inhibited by blocking viral replication. For VSV, the trigger for the switch from primary transcription to replication is the increased intracellular concentration of the viral nucleoprotein (N) (33, 34). In line with this concept, treatment with the translation inhibitor cycloheximide (CHX) is known to interfere with the switch from viral transcription to replication. When we treated HEK 293 cells with CHX 30 min prior to infection, viral protein synthesis was blocked and so was synthesis of immunostimulatory RNA (**Figures 2A–C**). Taken together these results argue that an essential part of the immunostimulaory RNA produced during infection with VSV is a result of replication after the VSV polymerase has switched from transcription to generation of progeny genomes.

### RIG-I-Associated RNA After VSV Infection Is Immunostimulatory and Contains Base-Paired RNAs With 5’-triphosphate Modifications

**Figure 3:**
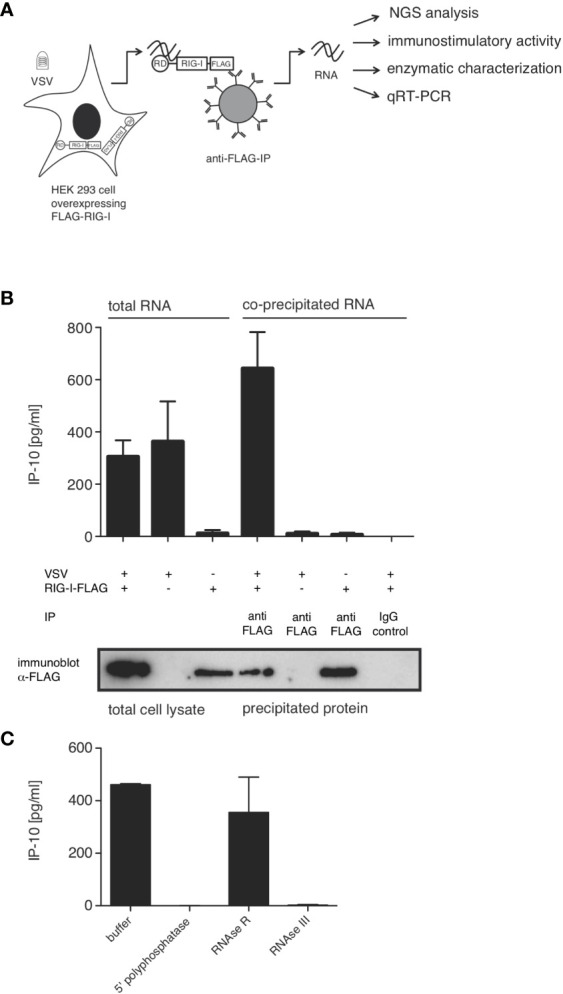
RIG-I-associated RNA after VSV infection is immunostimulatory and contains base-paired RNAs with 5’-triphosphate modifications. HEK 293 cells (40 x 10^6^) expressing FLAG-RIG-I or not were infected with VSV (MOI=1) or left uninfected. 9 hours later lysates were prepared and either used directly for analysis by SDS-PAGE and RNA isolation or immunoprecipitated with anti-FLAG antibody-coupled sepharose beads or beads coupled to an unspecific IgG-isotype control. After elution of the protein/RNA complexes from the beads, co-immunoprecipitated RNA was purified from the eluate and further analyzed as indicated. Aliquots of the immunoprecipitated proteins were analyzed by SDS-PAGE. **(A)** Schematic representation of the experimental set-up. RD (regulatory or repressor domain) the domain of RIG-I binding to the 5’-end of triphosphate modified RNA; also called c-terminal domain (CTD). **(B)** RNA isolated directly from infected cells or after immunoprecipitation was retransfected into 1205Lu melanoma cells (8 x 10^3^ cells/well) using RNAiMax Lipofectamin. IP-10 was measured in the supernatant after 24 hours by ELISA. In the lower panel western blot results from the corresponding lysates are depicted after staining with anti-FLAG antibodies. **(C)** RNA was treated with the indicated enzymes prior to retransfection into 1205Lu melanoma cells. IP-10 was measured in the supernatant after 24 hours. Data are shown as mean +- SD from n=2 independent experiments.

In order to characterize the RNA species triggering RIG-I during VSV infection, we co-immunoprecipitated FLAG-tagged RIG-I and the RNAs bound to it 9 hpi. When the co-immunoprecipitated RNA was isolated and retransfected into 1205Lu melanoma cells, RNA from infected, FLAG-RIG-I over-expressing cells was capable of inducing IP-10. Neither RNA co-immunoprecipitating with RIG-I in uninfected cells nor RNA from infected cells co-immunoprecipitating with control beads, nor RNA co-immunoprecipitating with FLAG-beads from infected cells lacking overexpressed FLAG-RIG-I showed immunostimulatory capacity (**Figures 3A, B**).

We and others have shown that optimal synthetic ligands for RIG-I consist of double-stranded 5’-tri- or 5’-di-phosphorylated RNA (11, 14, 15). In order to determine whether this also holds true for RNA bound to RIG-I originating from VSV, we treated the RNA co-immunoprecipitating with RIG-I with RNA-modifying enzymes prior to retransfection into 1205Lu cells. 5’-polyphosphatase removing tri- and diphosphate groups from the 5’ end of RNA and the double-strand specific RNase III abolished immunostimulatory activity, whereas single-strand specific RNase R had no effect on the induction of IP-10. This allows the conclusion that RIG-I-triggering RNA in VSV infection is at least partly double-stranded and contains a 5’-tri or di-phosphate group, both of which are required for immunostimulation (**Figure 3C**). These data are further evidence that the RNA co-purified with RIG-I indeed acts as a RIG-I ligand and not as MDA5- or TLR3-ligand as in these cases the immunostimulatory activity would be unaffected by the removal of the 5’-triphosphate group.

### Sequencing of RIG-I Associated RNAs After VSV Infection Reveals a 4719 Nucleotides Long Panhandle-Defective-Interfering-Genome and the Full-Length VSV Genome as Enriched Ligands for RIG-I

To characterize the RNA co-immunoprecipitating with RIG-I after VSV-infection in more detail we analyzed the RIG-I associated RNA by next generation sequencing. We applied a protocol for cDNA library preparation that encompasses sequential ligation of 5’and 3’ adapters to the RNA molecules, thus preserving the information of strand-specificity during the subsequent next generation sequencing run.

When we sequenced the total input RNA (total RNA/input RNA) after depleting ribosomal RNA, about 33% of the reads could be aligned to the VSV genome and 51% of RNAs could be aligned to the human genome with 16% unmapped reads. Similar numbers were found for RNAs co-immunoprecipitating with RIG-I (FLAG-RIG-I IP). However, also in the condition controlling for unspecific binding of RNA to FLAG-beads (w/o FLAG-RIG-I IP) a significant amount of RNA was recovered showing a pattern with 24% (only 6% less than in the RIG-I condition) of reads matching the VSV genome and 52% aligning to the human genome (see **Supplementary Figure S2**).

**Figure 4:**
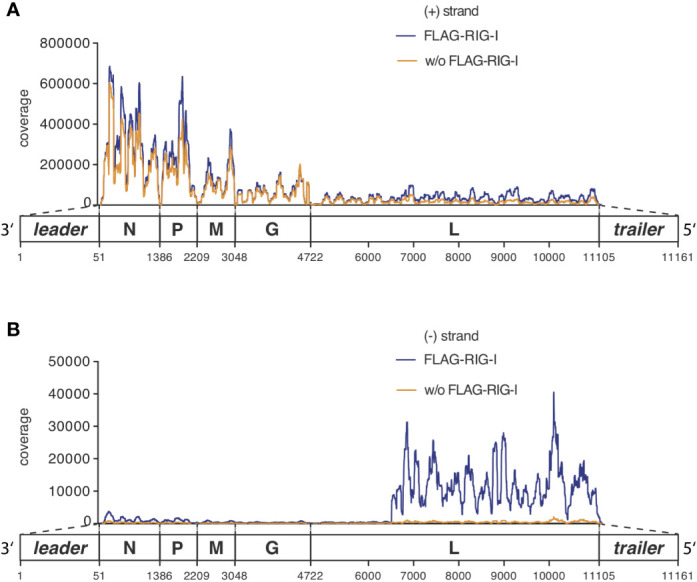
RNAs co-immunoprecipitated with anti-RIG-I-coated sepharose beads from lysates of VSV-infected cells consist mostly of unspecifically bound viral and endogenous mRNA transcripts but are specifically enriched for viral genomic sequences of negative orientation. HEK 293 cells (40 x 10^6^) expressing either FLAG-RIG-I or not were infected with VSV (MOI=1). 9 hours later lysates were prepared and RIG-I/RNA complexes were immunoprecipitated with anti-FLAG antibody-coupled sepharose beads. After elution of the protein/RNA complexes from the beads, RNA was purified from the eluate and used to generate cDNA libraries for next generation sequencing on an Illumina genome analyzer. The read sequences were aligned to the VSV genome reference in **(A)** sense or **(B)** antisense orientation and are depicted on the y-axis as coverage meaning the number of sequence reads that cover each specific position of the reference genome represented on the x-axis. The x-axis has a resolution down to single nucleotide positions. A schematic representation of the VSV genome underlines the x-axis and is in areas of the trailer and leader sequences not true to scale. One representative experiment out of two is shown.

In all three conditions the vast majority of RNAs of viral origin aligned in positive orientation to the reference genome (see **Supplementary Figure S2** and **Figure 4**). Positively oriented reads can either derive from mRNAs or the antigenome. The observation that the coverage decreases to almost zero at intergenic borders shows that most of the reads came from mRNAs and only very few reads in positive orientation do bridge neighboring genes as expected for antigenomes. A second pattern that matches the expectation for viral mRNAs of negative-stranded RNA viruses is the observed gradient in positive oriented reads with high coverage of genes at the 3’ end that decreases for genes at the 5’end (coverage of N > P > M > G > L). This gradient can be clearly visualized if the sequencing reads are not aligned to the VSV reference sequence in a resolution of single nucleotides but in separate windows pooling all sequences matching one of the gene segments (leader, N, P, M, G, L, trailer, see **Supplementary Figure S3**). The reason for this 3’ to 5’ gradient originates from the viral polymerase that starts transcription always at the promoter region of the 3’end and continues transcription of mRNAs with a certain probability of dropping from its template at each gene boundary (35). Remarkably the 3’leader and 5’ trailer regions were underrepresented in the sequence reads.

Looking specifically at the RNA from the RIG-I pulldown experiment and comparing it to the corresponding FLAG-bead control IP, both conditions were indistinguishable in their pattern of positively oriented RNAs caused by the mRNA gradient (**Figure 4A** and **Supplementary Figure S3**). We therefore concluded that these reads represented RNAs that unspecifically bound to the anti-FLAG-beads.

The full-length genome should theoretically be the only negatively-oriented RNA produced in VSV-infected cells. In alignments of the negative reads we therefore expected an even coverage over the whole reference genome. However, looking at our data we observed that this only applied until position 6496 where a sudden increase in coverage downstream to this position was seen in the input RNA as well as in RIG-I-associated RNAs that was lacking in the RNA recovered from the control IP (**Figure 4B**).

**Figure 5:**
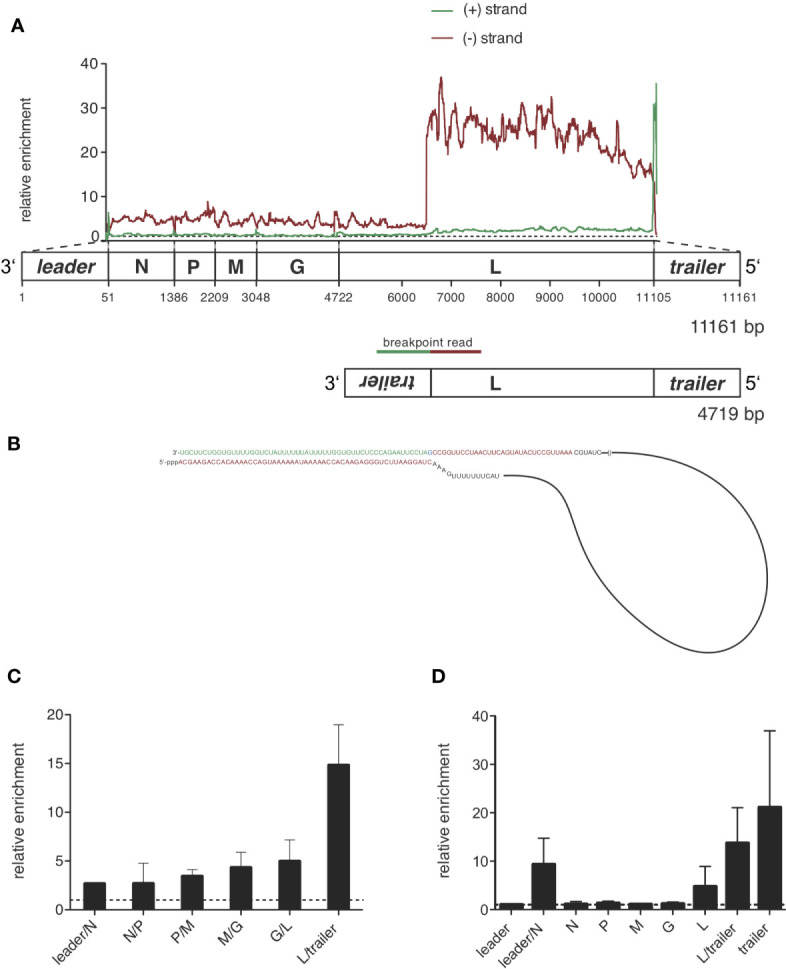
Sequencing of RIG-I-associated RNAs after VSV infection reveals a 4719 nucleotides long panhandle defective-interfering genome and the full length VSV genome as specific ligands bound to RIG-I. HEK 293 cells (40 x 10^6^) expressing either FLAG-RIG-I or not were infected with VSV (MOI=1). 9 hours later lysates were prepared and RIG-I/RNA complexes were immunoprecipitated with anti-FLAG antibody-coupled sepharose beads. After elution RNA was purified from the eluate. **(A)** cDNA libraries were generated and sequenced on an Illumina genome analyzer. The read sequences were aligned to the VSV genome reference and the coverage, meaning the number of sequence reads that cover each specific position of the reference genome represented on the x-axis was determined. Data show the relative enrichment on the y-axis by calculating the ratio of the coverage at each position in samples containing RIG-I divided by the coverage in the negative control without RIG-I. The x-axis has a resolution of single-nucleotide positions. A schematic representation of the VSV genome underlines the x-axis and is in areas of the trailer and leader sequences not true to scale. The analysis was performed separately for sequences aligning to the VSV genome in positive orientation (green) and negative orientation (red). One representative experiment out of two is shown. **(B)** Model of the detected 4719 nucleotides long panhandle DI genome. Breakpoint-containing reads covering the L-gene in negative orientation and the inverse complementary trailer sequence were found in the NSG libraries and are highlighted in red and green as basis for the DI genome model. **(C)** Depicted is the relative enrichment (coverage in samples containing RIG-I/coverage in the negative control without RIG-I) of NGS reads covering intergenic sequences between two genes in negative orientation. These sequences are uniquely contained in the genomic sequence of VSV. Data are shown as the mean + SD of two independent experiments. No leader/N intergenic read was detected in the control of one of the experiments. To avoid zero as the divisor here only n=1 is given. **(D)** RNA from the eluate was transcribed into cDNA and specific primer sets were used to detect the indicated genomic regions by RT-qPCR. The viral RNAs were normalized to the unspecifically bound HPRT sequences and are depicted as relative enrichment (copies/HPRT reads in samples containing RIG-I divided by copies/HPRT reads in the negative control without RIG-I). Data are shown as mean plus SD of n=1 (leader, M, G), n=2 (L-trailer) or n=3 (Leader-N, N, P, L, trailer).

In order to visualize the sequences specifically enriched through their binding to RIG-I we calculated a ratio called *relative enrichment* by dividing the number of reads in the FLAG-RIG-I pulldown by the number of reads in the control IP derived from cells that do not express FLAG-RIG-I. What we observed when we plotted this ratio was a small enrichment for negatively oriented reads from position one to position 6494. From there onwards however, the ratio in coverage increased to more than 20-fold (**Figure 5A**; **Supplementary Figures S3, S4**). Interestingly, calculating the ratio also revealed that the 3’ leader region and the 5’ trailer region were enriched between the two conditions in a sense-specific manner (**Figures 5A, C** and **Supplementary Figure S3D**). In the leader region, a small enrichment of the whole region in negative orientation (leader, leader/N) was complemented by a narrow peak in plus-orientation around nucleotides 44 – 61 spanning the leader/N junction (**Figure 5A**, **Supplementary Figure S3D**). A stronger enrichment for the trailer region could be seen in both orientations, which can be explained best by a RIG-I ligand containing a double-stranded trailer region.

The enrichment of negative reads at the rear part of the genome and the enrichment of the trailer region in both orientations fits a pattern that is typical for a copy-back defective interfering (DI) genome.

DI RNAs are truncated forms of the viral genomes generated by many viruses including VSV during viral replication [reviewed in (36, 37)]. As critical parts of the viral genome are missing, DIs are unable to replicate on their own and need a co-infecting complete virus as a “helper virus” to replicate. The generation of DI genomes is often explained by the “copy-choice” mechanism, which is caused by the error-prone viral polymerases. The polymerase skips during replication from one template to another, or from one part of the template to other parts, and resumes elongation at the 3′ end of the nascent chain it started to synthesize before it “skipped” (38, 39). Depending on their shape and the amount of the genome missing, DI genomes are categorized as copy-back/panhandle, snap-back/hairpin, or internal deletion DIs (36, 37).

The copy-back defective interfering particle identified here consists of approximately the last 4000 nucleotides of the genome with the trailer region being attached to its 3’-end in reversed orientation leading to a panhandle with a double-stranded trailer region.

To prove that this enrichment of trailer and L-gene sequences is due to a defective interfering genome bound to RIG-I, we searched for reads that would be specific for the proposed DI genome. These reads should cover the breakpoint between the position in the L gene at approximately position 6500 and the reverse trailer attached to it. Applying an algorithm looking for hybrid reads that partly aligned to the reference in forward and reverse orientation, we indeed found breakpoint reads covering both the trailer region starting at position 11 107 in positive orientation and the L gene from position 6496 in negative orientation. We could find 18 of these reads in the FLAG-RIG-I pulldown but none in the control condition. We could not find any other breakpoint reads in our libraries showing a coverage higher than 3 reads or enrichment between the pulldown-conditions. From this breakpoint-analysis we could deduce the sequence of the 4719 nucleotides long DI genome that is schematically depicted in **Figure 5B** and contains a panhandle with a 55-nucleotides double-stranded trailer region.

DI genomes are replicated similar to the whole genome *via* the production of an anti-DI genome that is then used as a template to produce new DI genome copies. As the sequence of the double-stranded stretch of the panhandle is identical for the identified DI genome and its DI-anti-genome it is not surprising that we found the sequence of the DI genome enriched not only in negatively oriented reads (DI genome) but also in positively oriented reads (anti-DI genome). The latter however in reduced amounts (**Figure 5A** and **Supplementary Figure S4**). An explanation for this might be that the genomic DI-genome is more abundant than the DI-antigenome at the specific time point we set up our experiment or that positive oriented anti-DI-genome sequences are concealed by the abundance of the overlapping positive oriented L-gene mRNAs found in both, the RIG-I IP as well as in the control condition.

Besides the strong enrichment of DI genomes in RIG-I-associated RNA we also saw a smaller enrichment of negatively oriented reads covering the rest of the viral genome not included in the identified DI genome, suggesting that the complete viral genome as a whole might also bind to RIG-I (**Figure 5A** and **Supplementary Figure S3D**). This could also be seen when we analyzed negatively oriented reads covering the area between two genes and between leader/trailer and the adjacent gene respectively. In these intergenic reads derived from the genome we saw a small but - with the exception of the L-trailer junction that is also included in the DI genome - equally distributed enrichment consistent with the binding of the whole VSV genome to RIG-I (see **Figure 5C**).

The same intergenic reads in positive orientation are only contained in the antigenome or aberrant mRNA-read-through sequences but not in normal mRNA- or leader-transcripts. Analyzing these positive oriented reads we only saw a small enrichment for leader/N junction reads but not for the intergenic reads of the N/P, P/M, M/G and G/L junction arguing that the antigenome as a whole is not relevantly bound and enriched by RIG-I (**Supplementary Figure S3D** and data not shown).

To verify the next generation sequencing results with a second method we additionally analyzed RIG-I-bound RNA using qPCR. We designed qPCR assays for every VSV gene, the trailer region and assays covering the intergenic region between the leader region and the N gene and between the L-gene and the trailer region. As we used random hexamer primers for reverse transcription these assays however cannot distinguish between positively and negatively oriented RNAs. RNA eluted from the used anti-FLAG beads in both conditions, the Flag-RIG-I containing and the control condition not containing FLAG-RIG-I, to our initial surprise contained similar amounts of unspecifically bound HPRT-mRNA. This unspecific binding however, could be used to normalize the amount of the viral RNAs to the bound HPRT-mRNA.

The qPCR data confirmed the binding of DI genomes to RIG-I seen by the NGS data. Similar to the NGS data the quantitatively most abundant viral RNAs were VSV mRNAs detected by the qPCR assays covering the VSV genes. Calculating the *relative enrichment* called ratio dividing the copies/HPRT in the FLAG-RIG-I pulldown by the copies/HPRT in the control IP derived from cells that do not express FLAG-RIG-I, the L-region, the trailer assay and the leader-N assay were clearly enriched compared to the control (see **Figure 5D**). The enrichment seen in the L-, L/trailer- and trailer-assay is thereby explained by the identified DI genome. As the gene-specific PCR assays do not differentiate between the genomic sequences and mRNA sequences the enrichment of the whole genome in negative orientation seen in the NGS reads is covered by the vast number of mRNAs detected by the same qPCR assays, but might at least in part explain the enrichment of RNAs in the leader/N assay.

### DI-Depleted VSV Stocks Lose Most of Their Immune-Stimulating Capacity but Retain Whole Genome and Leader-N-Read-Through Containing RNA-Sequences as Trigger for RIG-I

To test the extent to which the identified VSV-DI genome is responsible for triggering the RIG-I-dependent immune activation we generated VSV stocks devoid of DI genomes.

A defective interfering particle lacks parts of the genome necessary to perform a complete replication cycle and therefore can only replicate in a cell if the cell is simultaneously infected by a complete virus that provides the missing genetic information for the replication machinery. This allows diluting out DI genomes from virus stocks by passaging the virus-stocks using very low multiplicities of infection (MOI).

**Figure 6:**
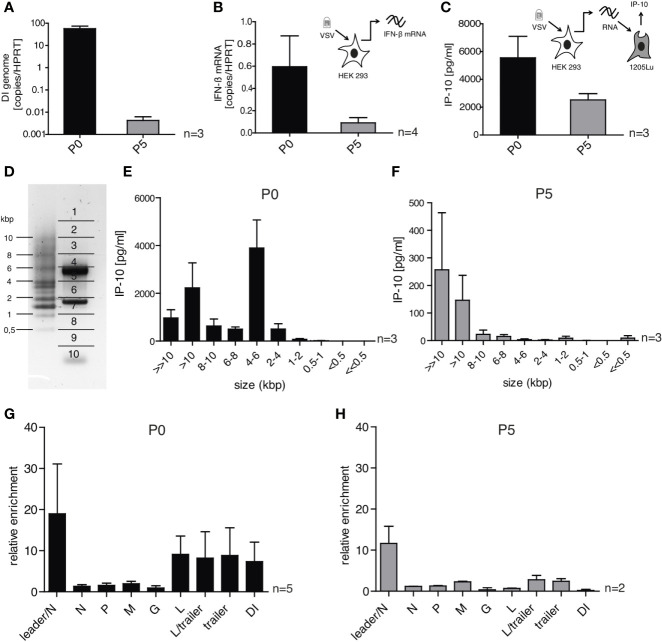
DI-depleted VSV stocks lose most of their immune-stimulating capacity but retain whole genome and leader-N read-through containing RNA sequences as trigger for RIG-I. HEK 293 cells were infected with VSV (MOI =1) using virus stocks containing either high (P0) or low (P5) amounts of DI genomes. 24 h post infection the infected cells were lysed and their RNA was isolated. **(A, B)** The isolated RNA was transcribed to cDNA and was analyzed by RT-qPCR for **(A)** the amount of DI genomes and **(B)** the expression of IFN-β. **(C)** The isolated RNA was retransfected into 1205Lu cells (100 ng/100 µl) using RNAiMax lipofectamin. IP-10 in the supernatant was measured 24 h after transfection by ELISA. **(D)** The isolated RNA was subjected to size-dependent separation on an agarose gel and divided into ten slices as indicated. **(E, F)** The recovered RNA of each slice was used for retransfection into 1205Lu cells and IP-10 in the supernatant was measured 24 h after transfection. **(G, H)** FLAG-RIG-I immunoprecipitation was performed 24 h post infection with VSV from the P0 or P5 stock in HEK 293 cells either expressing FLAG-RIG-I or not. The co-precipitated RNA was isolated and analyzed by quantitative RT-PCR using gene- and DI-specific primers. Enrichment for each genomic region was calculated as ratio of the relative amount (copies/HPRT reads) in immunoprecipitated RNA of the FLAG-RIG-I-overexpressing cells and the FLAG-RIG-I-lacking cells. Data are represented as mean ± SEM of n=2-5 independent experiments as indicated.

Starting with our DI-containing virus stock called P0, BHK cells were infected at an MOI of 10^-6^ and after 24 h the supernatant was collected and reused for a repetitive infection each time with an MOI of 10^-6^. This procedure was repeated for 5 passages until in the resulting stock called P5 the DI genome was nearly completely diluted out as confirmed by RT-qPCR using a DI-specific qPCR assay, that is based on the unique sequence of the breakpoint between position 6496 and position 11 107 (**Figure 6A**). The stock depleted of DI genomes (P5) showed a greatly reduced induction of IFN-β measured 24 hpi by RT-qPCR (**Figure 6B**). This was in line with a decreased immunostimulatory capacity of the total RNA isolated at 24 hpi from cells infected with P5 compared to cells infected with P0 and used for retransfection into uninfected 1205Lu cells. In this assay that measures the transferable amount of immunostimmulatory RNA in a cell, the IP-10 amount in the supernatant 24 h post transfection was decreased by about 50% when the RNA was isolated from cells infected with the P5 stock compared to cells infected with the P0-stock (**Figure 6C**).

These results are in accordance with the hypothesis that DI genomes are the main, even though not the only trigger of the interferon responses after VSV infection in our original infection assays with P0. Accordingly, we found the production of DI genomes in infected cells to follow similar kinetics as the IFN-induction and the production of transferable stimulatory RNA in P0-infected cells. In addition, DI genome production was fully abolished by translational blockade through CHX (see **Supplementary Figure S5A** and **Figures 1**, **2**).

To further allocate the immunostimulatory capacity to specific RNA species we correlated the size and immunostimulatory capacity of RNAs after infection with VSV and analyzed the differences between the viral stocks P0 and P5 in this respect.

To do this, the RNA of HEK 293 cells infected with either P0 or P5 was isolated and fractionated into ten fractions on an agarose gel according to size (**Figure 6D**). PCR assays for the DI genome (4719 bp) and the N protein mRNA (1326 bp) found peaks for these RNAs in the expected fractions confirming the validity of the size-fractionation (**Supplementary Figures S5C, D**). The RNA of each fraction was then isolated and retransfected into 1205Lu cells to test their immunostimulatory capacity measured by the ability to induce the secretion of IP-10 (**Figures 6E, F**). The overall strongest IP-10 induction was observed in the fraction containing RNA molecules of 4 – 6 kbp for the P0 stock (**Figure 6E**). This fits to the size of the detected DI genome (4719 bp) and as expected, the immunostimulatory capacity of this fraction was gone in the stock with low levels of DI genomes (P5) (**Figure 6F**). RNAs of the fractions containing RNA molecules in the range of the full-length genome (8-10kbp and >10 kbp) showed induction of IP-10 for both viral stocks P0 and P5 although the total amount of IP-10 differed between these stocks.

When we analyzed RNA co-precipitating with RIG-I after infection with VSV in the size-fractionation assay we could replicate the size-profile of immunostimulatory RNAs seen above using complete cellular RNA arguing that the immunostimulatory activity is indeed mediated *via* RIG-I (see **Supplementary Figure S5B**). Comparing RNA sequences co-immunoprecipitating with RIG-I after infection with P0 and P5 respectively using our qPCR assays we found a similar enrichment in both conditions in the leader/N assay that detects the full-length genome or aberrant so-called Leader/N readthrough transcripts. The enrichment in the DI genome specific PCR assay seen in P0 however was gone in P5 and the same was true for the L-, L/trailer-, and trailer-sequences indicating that most of the enrichment in these sequences resulted from the DI genome (**Figures 6G, H**).

In summary, the data clearly show that in infections with our standard stocks of the VSV wt the DI genome has been the main activator of RIG-I. In the absence of DI genomes however, other leader/N containing VSV-sequences such as the full-length genome and mRNA read-throughs are still able to trigger RIG-I and induce an interferon response even though to a lesser extent.

In order to replicate despite the interferon signaling triggered by RIG-I, VSV has evolved strategies to evade that cellular response using its M-protein to shut down host gene expression [reviewed in (25, 40)]. The M-protein localizes in host cells to the nuclear envelope and competes with cellular RNA for binding to cellular Rae1 and Nup98 through its methionine at position 51, thereby inhibiting nucleocytoplasmic trafficking of cellular mRNAs and small nuclear RNAs. The inhibition of nuclear RNA transport prevents host gene expression and suppresses the production of IFNs and other antiviral proteins in infected cells (41, 42). Substitution of methionine by arginine at position 51 (M51R mutation) makes the M-protein unable to inhibit host gene expression, while it still remains intact for its other functions in virus assembly. The VSV M51R mutant was found to be defective in its ability to block host gene expression and therefore, able to induce higher IFN signaling in responsive cells (43, 44). In an attempt to improve safety and as a strategy to induce stronger virus-mediated antitumor immune responses the VSV-M51R variant has been proposed as an improved basis for oncolytic applications (6, 25).

We therefore tested, if the increased IFN-responses described for the VSV M51R mutant compared to the wildtype are RIG-I-dependent and similarly driven or enhanced by DI-genomes. While the amount of IP-10 induced in the first 24 h by infection with the VSV M51R mutant in 1205Lu cells exceeded the amount induced by VSV wt by up to 100-fold, there was no difference in its dependence on RIG-I and MAVS without major contributions by MDA5 or PKR (see **Supplementary Figures S6 **and** S1**). Using a single primer PCR-strategy that applies only one primer binding to the trailer region we could amplify products in our VSV M51R stocks indicating the presence of sequences typical for copy back DI-genomes that contain the trailer sequence twice, in forward- as well as in reverse complementary orientation. Sequencing of the amplified PCR products revealed a copyback DI genome of 3159 bp length with a breakpoint at genomic position 8103 (DI 8103). The same approach when applied to our VSV wt stocks reconfirmed the presence of the 4719 bp long DI genome with its breakpoint at position 6494 (DI 6494) we had already identified by next generation sequencing (see **Supplementary Figure S7A**). The identified breakpoint at position 8103 in the DI from the VSV M51R stock allowed to design an RT-qPCR assay for specific quantification of the DI 8103 (see **Supplementary Figure S7B**). Similar to VSV wt repetitive passaging of the original VSV M51R stocks (P0) with very low MOI could generate a stock almost completely devoid of the DI 8103 genomes (P3) (see **Supplementary Figure S7C**). The VSV M51R stock depleted of DI genomes (P3) lost almost all its ability to induce IP-10 in the first 24 h after infection of Hek293 cells (see **Supplementary Figure S7D**). This was in line with a greatly decreased RIG-I-dependent immunostimulatory capacity of the total RNA isolated at 24 hpi from cells infected with M51R (P3) compared to cells infected with M51R P0 and used for retransfection into uninfected 1205Lu wt or RIG-I-deficient cells (**Supplementary Figure S7E**). Interestingly, in contrast to the 100-fold difference in IP-10 production after infection of 1205Lu wt cells with VSV wt P0 and VSV M51R, in the RNA transfer assay that relies purely on the transfer of RNA and excludes effects of the VSV-M protein, RNA derived after infection with VSV wt induces similar amounts of IP-10 as RNA-derived after infection with the M51R mutant (see **Supplementary Figures S1, S6 **and** S7E**). Together these data confirm that the importance of DI-genomes as drivers of the RIG-I-dependent interferon response apply to both, the VSV wt and the M51R mutant strain.

## Discussion

The identification of double-stranded triphosphate RNA as the PAMP detected by RIG-I posed the question which RNA species in the replication cycle of the different RIG-I-restricted viruses are the relevant, naturally occurring ligands. Since then, many putative ligands have been described (13, 16–18, 45, 46). Yet, although extensively studied as a model for mononegavirales biology and being developed as an instrument for vaccines and oncolytic therapy, the RIG-I ligands occurring during infection with VSV have not been clarified. The present study addressed this question by sequencing RNA that co-immunoprecipitated with RIG-I after VSV-infection, an approach that has previously been employed successfully to identify RIG-I-bound RNA during Sendai, influenza and measles virus infection (16, 17).

Enzymatic treatment of RNA that co-immunoprecipitated with RIG-I confirmed that the recognition of VSV-RNA relies on 5’-tri- or di-phosphates and double-strandedness. Therefore, interferon-inducing RNA during VSV infection meets the canonical structural requirements that have been determined to be essential for RIG-I activation (12, 14, 15). Analyzing the RNA purified *via* RIG-I immunoprecipitation revealed the binding of a novel 4.7 kb copy-back defective interfering genome. Despite a distinct break-point at position 6494, the composition of this DI genome, and the DI 8103 identified in our VSV M51R stocks with their complementary trailer-regions resemble previously described copyback defective-interfering genomes of VSV, such as DI-T, DI-T(L) and DI 611 (47–49) (see **Supplementary Figure S8**). The specific structure and number of DI-genomes in a given VSV virus stock is to some extent the product of chance and will vary between different labs and stocks. However, based on data from other negative-strand RNA viruses (50), the genesis of DI-genomes is likely to be a regular event as it already happens in early passages of recombinantly generated viruses and based on our experience passaging even at low MOIs of 0.1-0.001 can inadvertently lead to DI-containing stocks.

The reduction of DI content in cellular extracts of VSV-infected cells resulted in a reduced yet not fully abolished immunostimulatory capacity.

This raised the question what the nature of immunostimulatory RNA in the absence of DI genomes could be. Surprisingly, the most abundant reads in our pull-down experiments were of positive orientation and their alignment showed the classical gradient of VSV mRNAs (35, 51). Yet, these reads were not enriched between the FLAG-RIG-I-pull-down and the control. We therefore exclude the possibility that mRNAs are a trigger of RIG-I. Instead we found evidence for the interaction of full-length genomic RNA with RIG-I: the alignment of negative oriented reads depicted a discrete but consistent enrichment over the whole genome in RIG-I immunoprecipitations. This was further corroborated by the quantification of negative-strand-specific reads crossing viral gene boundaries of the VSV genome.

In line with the NGS data, size-fractionation experiments revealed that immunostimulatory RNA isolated from VSV-infected cells migrated at the predicted size of the novel DI genome (4.7 kb) but also of the full-length genome (11 kb). When cell extracts from cells infected with virus stocks with reduced DI content were analyzed, only the RNA migrating at the size of the full-length genome retained immunostimulatory properties. Together these data suggest, that the full-length genome can activate RIG-I in the absence of DI genomes.

Interestingly, we also detected an enrichment of leader-N read-through sequences in our RIG-I pulldown experiments which were detectable both in qPCR and NGS. According to the NGS data, the enriched sequence spanning the leader/N junction is of positive orientation, while the qPCR assay cannot distinguish positive from negative oriented RNA. However, while one would expect leader-N-read-through products to migrate at sizes below 0.5 kb in the size fractionation experiment, RNA isolated from this fraction was not immunostimulatory. Therefore, we consider it possible that the leader-N-read-through transcript is only immunostimulatory when attached to the full-length genome, thereby forming a double-strand that can be detected by RIG-I.

The enrichment of leader-N in positive polarity has been also observed in next generation sequencing analyses of RIG-I-bound RNA after measles virus infection (17). The VSV leader RNA is the first to be transcribed, lacks a cap and is immunogenic when transcribed *in vitro* using the recombinant VSV L protein (52). The coverage for the leader region in our experiments was extremely low, contrasting reports that claim that the leader RNA is the most abundant RNA in VSV infected cells. This discrepancy could be attributable to technical reasons such as our PCR assay not reaching adequate efficiency, the underrepresentation of 3’ and 5’ ends in NGS data or to the fact that the leader RNA shuttles quickly to the nucleus to alter host transcription (53) and we isolated cytoplasmic RNA rather than nuclear. Of note, even though the use of *in vitro* transcribed RNA has been instructive to delineate the nature of RIG-I ligands, it was the propensity of the T7 polymerase to form unexpected, partially double-stranded copy-back RNA that allowed to uncover the structure of RIG-I ligands (15). Intriguingly, the unintended copy-back byproducts of the viral T7 polymerase are reminiscent of panhandle defective interfering RNAs generated by the RNA dependent RNA polymerases (RdRp) of mononegavirales.

This study adds a quite prominent model virus to the panel of viruses (17), that have been described to elicit RIG-I signaling *via* a defective interfering genome. Furthermore, we find evidence for its full-length genome engaging with RIG-I. Both - the DI- and full-length genomes - are tightly packed into the nucleocapsid and this is thought to preclude the formation of RNA double-strands. This raises the question how RIG-I can access the RNA and what the nature of the double-strand is. There are several potential explanations for these questions:

Firstly, as it was uncovered recently that RIG-I itself exerts an effector-like function and is capable of displacing viral proteins from double-stranded RNA, it could be a RIG-I-intrinsic function to access RNA even when covered by ribonucleoprotein (54).

Secondly, double-stranded RNA can arise when the production of viral RNA exceeds the supply of N protein. Especially in the case of DI genomes this is a plausible scenario. The RNA from DI genomes - as explained above - has a replication advantage but does not code for a single mRNA. Therefore, it does not contribute to the supply of viral proteins potentially leading to an excess of genomes over N protein.

Thirdly, during transcription and replication the N protein wrap gets disassembled at least partially and temporarily to allow for the L protein to bind and synthesize the complementary strand.

In our experiments, we observed that replication of viral genomic RNA requires a constant supply of fresh proteins. Interrupting this supply with cycloheximide abolished synthesis of immunostimulatory RNA. This observation is consistent with previous reports (32) but in sharp contrast to others (18), in which interferon induction in VSV-infected cells could be observed even in the presence of cycloheximide. Differing concentrations of DI genomes in the viral stocks used for infection are a possible explanation for these discrepancies. We assume that VSV stocks with a very high DI content can elicit an interferon response even in the presence of translation-inhibition as shown for paramyxovirus. In experiments in which cycloheximide was not capable of blocking the emergence of immunostimulatory RNA in paramyxovirus-infected cells, a 19- to 59-fold excess of DI-RNA over full-length genomes was reported (55, 56). Based on our next-generation-sequencing data of total RNA from VSV-infected cells, the coverage of negatively oriented reads after the DI-break-point (towards the 5’end) was only 5.9-fold higher than the coverage towards the 3’-end of the genome, hinting at a moderate excess of DI genome over full-length genome.

In contrast to long-standing concerns that DI particles are mere cell-culture artifacts of high MOI passaging, there is now increasing evidence that DI genomes constitute an important source of immunogenic RNA in negative strand RNA virus infection, that actually arise *in vivo* and fine tune the outcome of viral infection and persistence (36). DI genomes could be detected in the nasopharyngeal fluid of children infected with RSV and correlated with the induction of antiviral genes. In addition, in a mouse model only mice infected with DI-rich virus stocks mounted an antiviral immune response and in consequence showed less severe lung pathology upon infection with RSV (57). Furthermore, defective interfering RNA genomes could be isolated from clinical samples of a broad range of pathogenic RNA viruses including measles, hepatitis A to C, dengue, influenza A, and respiratory syncytial virus [recently reviewed in (36)].

Many negative strand RNA viruses encode proteins that antagonize antiviral responses. As explained above, in VSV this is mediated by shutdown of host gene expression by the VSV matrix (M) protein (40). It is well conceivable that this suffices to block the interferon response to a weak or less abundant RIG-I agonist such as the full-length genome. Yet, when defective interfering RNAs are present, these truncated genomes are readily being replicated but do not contribute to protein synthesis of antagonists of antiviral signaling. As a result, the threshold of immune activation is being surpassed. In the M51R mutant the interferon response once triggered is not inhibited leading to a substantially higher amplitude of the response. However, even though shifted to a higher level still the same consequences of changes in the amount of upstream RIG-I ligands seen in VSV wt stocks apply. Consequently, as shown by our data, in the M51R mutant stocks the presence or absence of DI-genomes also strongly influences the strength of the induced immune response.

The fact that a VSV-based vaccine against EBOLA is already in clinical use, that the VSV-vaccine platform is currently used to develop a vaccine against COVID-19 and that VSV-strains are tested in clinical studies as oncolytic viruses for the treatment of tumors confers clinical relevance to our findings. The question how these therapeutic applications are being influenced by the content of defective interfering RNA in the viral preparations has not been addressed to date. In light of our findings this warrants further investigations. We assume that the DI content of a VSV-based vaccine strain or oncolytic virus will influence immunization success, anti-tumor immunity and side-effects. Depending on the specific context DI content will thereby enhance or reduce the wanted effect. We hope that awareness for the potential role of DI genomes as trigger of innate immune responses in VSV-based therapeutics will help to standardize DI genome content in virus stocks and might help to purposefully deplete or use DI genomes as natural adjuvants in VSV-based therapeutics.

## Data Availability Statement

The data presented in the study are deposited in the European Nucleotide Archive (ENA) repository, accession number PRJEB42788.

## Author Contributions 

AL and VB designed and performed experiments, analyzed results, and co-wrote the manuscript. SR initiated the project, designed the research, supervised and analyzed experiments and co-wrote the manuscript. AS and NL initiated the project, performed preliminary experiments, and gave conceptual advice. CB and MD performed bioinformatic analyses of the NGS data. GS, PS, FD, JR and DFRB performed experiments, contributed reagents and analyzed results. DP, JTS, LMK, HK, MS, SK, and SE supervised experiments and gave conceptual advice. All authors contributed to the article and approved the submitted version.

## Funding

This work was supported by *Einheit für Klinische Pharmakologie* (EKLIP), Helmholtz Zentrum München, Neuherberg, Germany (to SR, HK, SE, JTS), the international doctoral program “iTarget: Immunotargeting of cancer” funded by the Elite Network of Bavaria (to MS, LMK, SK and SE), the Marie-Sklodowska-Curie Training Network for the Immunotherapy of Cancer (IMMUTRAIN, grant number 641549) funded by the H2020 program of the European Union (SE, SK and MS), the Else Kröner-Fresenius-Stiftung 2017_A50 to LMK, the Friedrich-Baur-Stiftung to LMK, JTS and HK and the Deutsche Forschungsgemeinschaft KO50552-1 to SK, DFG - project number 442265435 to LMK, SCHN 664/6-1 and SFB 1321/P16 grant number 329628492 to MS, Ro 25257/-1 grant number 391217598 and SFB/TR-237-B14 Grant No. 369799452 –Projektnumber 404450088 to SR and funding through endowments to the medical faculty of the LMU to SR and HK.

## Conflict of Interest

The authors declare that the research was conducted in the absence of any commercial or financial relationships that could be construed as a potential conflict of interest.

## References

[1] Burrell CJ Howard CR Murphy FA. Chapter 27 - Rhabdoviruses. In: Burrell CJ Howard CR Murphy FA, editors. Fenner and White"s Medical Virology (Fifth Edition). London: Academic Press (2017). p. 383–94. 10.1016/B978-0-12-375156-0.00027-8

[2] Henao-Restrepo AM Camacho A Longini IM Watson CH Edmunds WJ Egger M. Efficacy and effectiveness of an rVSV-vectored vaccine in preventing Ebola virus disease: final results from the Guinea ring vaccination, open-label, cluster-randomised trial (Ebola Ca Suffit)! Lancet (2017) 389(10068):505–18. 10.1016/S0140-6736(16)32621-6 PMC5364328 28017403

[3] Medaglini D Santoro F Siegrist CA. Correlates of vaccine-induced protective immunity against Ebola virus disease. Semin Immunol (2018) 39:65–72. 10.1016/j.smim.2018.07.003 30041831

[4] Menicucci AR Jankeel A Feldmann H Marzi A Messaoudi I. Antiviral Innate Responses Induced by VSV-EBOV Vaccination Contribute to Rapid Protection. MBio (2019) 10(3):e00597–19. 10.1128/mBio.00597-19 PMC6538780 31138743

[5] Felt SA Grdzelishvili VZ. Recent advances in vesicular stomatitis virus-based oncolytic virotherapy: a 5-year update. J Gen Virol (2017) 98(12):2895–911. 10.1099/jgv.0.000980 PMC5845697 29143726

[6] Melzer MK Lopez-Martinez A Altomonte J. Oncolytic Vesicular Stomatitis Virus as a Viro-Immunotherapy: Defeating Cancer with a “Hammer” and “Anvil”. Biomedicines (2017) 5(1). 10.3390/biomedicines5010008 PMC5423493 28536351

[7] Kell AM Gale M Jr. RIG-I in RNA virus recognition. Virology (2015) 479-480:110–21. 10.1016/j.virol.2015.02.017 PMC4424084 25749629

[8] Kato H Takahasi K Fujita T. RIG-I-like receptors: cytoplasmic sensors for non-self RNA. Immunol Rev (2011) 243(1):91–8. 10.1111/j.1600-065X.2011.01052.x 21884169

[9] Kato H Takeuchi O Sato S Yoneyama M Yamamoto M Matsui K. Differential roles of MDA5 and RIG-I helicases in the recognition of RNA viruses. Nature (2006) 441(7089):101–5. 10.1038/nature04734 16625202

[10] Furr SR Moerdyk-Schauwecker M Grdzelishvili VZ Marriott I. RIG-I mediates nonsegmented negative-sense RNA virus-induced inflammatory immune responses of primary human astrocytes. Glia (2010) 58(13):1620–9. 10.1002/glia.21034 PMC2946392 20578054

[11] Goubau D Schlee M Deddouche S Pruijssers AJ Zillinger T Goldeck M. Antiviral immunity *via* RIG-I-mediated recognition of RNA bearing 5’-diphosphates. Nature (2014) 514(7522):372–5. 10.1038/nature13590 PMC4201573 25119032

[12] Hornung V Ellegast J Kim S Brzozka K Jung A Kato H. 5’-Triphosphate RNA is the ligand for RIG-I. Science (2006) 314(5801):994–7. 10.1126/science.1132505 17038590

[13] Pichlmair A Schulz O Tan CP Naslund TI Liljestrom P Weber F. RIG-I-mediated antiviral responses to single-stranded RNA bearing 5’-phosphates. Science (2006) 314(5801):997–1001. 10.1126/science.1132998 17038589

[14] Schlee M Roth A Hornung V Hagmann CA Wimmenauer V Barchet W. Recognition of 5’ triphosphate by RIG-I helicase requires short blunt double-stranded RNA as contained in panhandle of negative-strand virus. Immunity (2009) 31(1):25–34. 10.1016/j.immuni.2009.05.008 19576794 PMC2824854

[15] Schmidt A Schwerd T Hamm W Hellmuth JC Cui S Wenzel M. 5’-triphosphate RNA requires base-paired structures to activate antiviral signaling *via* RIG-I. Proc Natl Acad Sci U S A (2009) 106(29):12067–72. 10.1073/pnas.0900971106 PMC2705279 19574455

[16] Baum A Sachidanandam R Garcia-Sastre A. Preference of RIG-I for short viral RNA molecules in infected cells revealed by next-generation sequencing. Proc Natl Acad Sci U S A (2010) 107(37):16303–8. 10.1073/pnas.1005077107 PMC2941304 20805493

[17] Runge S Sparrer KM Lassig C Hembach K Baum A Garcia-Sastre A. In vivo ligands of MDA5 and RIG-I in measles virus-infected cells. PLoS Pathog (2014) 10(4):e1004081. 10.1371/journal.ppat.1004081 24743923 PMC3990713

[18] Weber M Gawanbacht A Habjan M Rang A Borner C Schmidt AM. Incoming RNA virus nucleocapsids containing a 5’-triphosphorylated genome activate RIG-I and antiviral signaling. Cell Host Microbe (2013) 13(3):336–46. 10.1016/j.chom.2013.01.012 PMC5515363 23498958

[19] Menon S Boyer NP Winkle CC McClain LM Hanlin CC Pandey D. The E3 Ubiquitin Ligase TRIM9 Is a Filopodia Off Switch Required for Netrin-Dependent Axon Guidance. Dev Cell (2015) 35(6):698–712. 10.1016/j.devcel.2015.11.022 26702829 PMC4707677

[20] Schmid-Burgk JL Schmidt T Gaidt MM Pelka K Latz E Ebert TS. OutKnocker: a web tool for rapid and simple genotyping of designer nuclease edited cell lines. Genome Res (2014) 24(10):1719–23. 10.1101/gr.176701.114 PMC4199374 25186908

[21] Lawson ND Stillman EA Whitt MA Rose JK. Recombinant vesicular stomatitis viruses from DNA. Proc Natl Acad Sci U S A (1995) 92(10):4477–81. 10.1073/pnas.92.10.4477 PMC41967 7753828

[22] Wollmann G Rogulin V Simon I Rose JK van den Pol AN. Some attenuated variants of vesicular stomatitis virus show enhanced oncolytic activity against human glioblastoma cells relative to normal brain cells. J Virol (2010) 84(3):1563–73. 10.1128/JVI.02040-09 PMC2812324 19906910

[23] Wu L Huang TG Meseck M Altomonte J Ebert O Shinozaki K. rVSV(M Delta 51)-M3 is an effective and safe oncolytic virus for cancer therapy. Hum Gene Ther (2008) 19(6):635–47. 10.1089/hum.2007.163 PMC2775926 18533893

[24] Ebert O Shinozaki K Huang TG Savontaus MJ Garcia-Sastre A Woo SL. Oncolytic vesicular stomatitis virus for treatment of orthotopic hepatocellular carcinoma in immune-competent rats. Cancer Res (2003) 63(13):3605–11. 12839948

[25] Hastie E Grdzelishvili VZ. Vesicular stomatitis virus as a flexible platform for oncolytic virotherapy against cancer. J Gen Virol (2012) 93(Pt 12):2529–45. 10.1099/vir.0.046672-0 PMC4091291 23052398

[26] Ruzicka M Koenig LM Formisano S Boehmer DFR Vick B Heuer EM. RIG-I-based immunotherapy enhances survival in preclinical AML models and sensitizes AML cells to checkpoint blockade. Leukemia (2020) 34(4):1017–26. 10.1038/s41375-019-0639-x PMC7214254 31740809

[27] Schmidt A Linder A Linder N Rothenfusser S. Isolation of RIG-I-associated RNAs from virus-infected cells. Methods Mol Biol (2014) 1169:37–44. 10.1007/978-1-4939-0882-0_4 24957227

[28] Gerlier D Lyles DS. Interplay between innate immunity and negative-strand RNA viruses: towards a rational model. Microbiol Mol Biol Reviews: MMBR (2011) 75(3):468–90. 10.1128/MMBR.00007-11 PMC3165544 21885681

[29] Besch R Poeck H Hohenauer T Senft D Hacker G Berking C. Proapoptotic signaling induced by RIG-I and MDA-5 results in type I interferon-independent apoptosis in human melanoma cells. J Clin Invest (2009) 119(8):2399–411. 10.1172/JCI37155 PMC2719920 19620789

[30] Carey BL Ahmed M Puckett S Lyles DS. Early steps of the virus replication cycle are inhibited in prostate cancer cells resistant to oncolytic vesicular stomatitis virus. J Virol (2008) 82(24):12104–15. 10.1128/JVI.01508-08 PMC2593309 18829743

[31] Lyles D Kuzmin I Rupprecht C. Rhabdoviridae. In: Knipe DMHP, editor. Fields Virology, vol. Vol 1. Philadelphia: Lippincott Williams & Wilkins, Wolters Kluwer Business (2013). p. 885–922.

[32] tenOever BR Sharma S Zou W Sun Q Grandvaux N Julkunen I. Activation of TBK1 and IKKepsilon kinases by vesicular stomatitis virus infection and the role of viral ribonucleoprotein in the development of interferon antiviral immunity. J Virol (2004) 78(19):10636–49. 10.1128/JVI.78.19.10636-10649.2004 PMC516426 15367631

[33] Patton JT Davis NL Wertz GW. N protein alone satisfies the requirement for protein synthesis during RNA replication of vesicular stomatitis virus. J Virol (1984) 49(2):303–9. 10.1128/JVI.49.2.303-309.1984 PMC255465 6319730

[34] Wertz GM Howard MB Davis N Patton J. The switch from transcription to replication of a negative-strand RNA virus. Cold Spring Harb Symp Quant Biol (1987) 52:367–71. 10.1101/SQB.1987.052.01.042 2841068

[35] Iverson LE Rose JK. Localized attenuation and discontinuous synthesis during vesicular stomatitis virus transcription. Cell (1981) 23(2):477–84. 10.1016/0092-8674(81)90143-4 6258804

[36] Manzoni TB Lopez CB. Defective (interfering) viral genomes re-explored: impact on antiviral immunity and virus persistence. Future Virol (2018) 13(7):493–503. 10.2217/fvl-2018-0021 30245734 PMC6136085

[37] Yang Y Lyu T Zhou R He X Ye K Xie Q. The Antiviral and Antitumor Effects of Defective Interfering Particles/Genomes and Their Mechanisms. Front Microbiol (2019) 10:1852. 10.3389/fmicb.2019.01852 31447826 PMC6696905

[38] Lazzarini RA Keene JD Schubert M. The origins of defective interfering particles of the negative-strand RNA viruses. Cell (1981) 26(2 Pt 2):145–54. 10.1016/0092-8674(81)90298-1 7037195

[39] Perrault J. Origin and replication of defective interfering particles. Curr Top Microbiol Immunol (1981) 93:151–207. 10.1007/978-3-642-68123-3_7 7026180

[40] Rieder M Conzelmann KK. Rhabdovirus evasion of the interferon system. J Interferon Cytokine Res (2009) 29(9):499–509. 10.1089/jir.2009.0068 19715459

[41] Ahmed M McKenzie MO Puckett S Hojnacki M Poliquin L Lyles DS. Ability of the matrix protein of vesicular stomatitis virus to suppress beta interferon gene expression is genetically correlated with the inhibition of host RNA and protein synthesis. J Virol (2003) 77(8):4646–57. 10.1128/JVI.77.8.4646-4657.2003 PMC152115 12663771

[42] von Kobbe C van Deursen JM Rodrigues JP Sitterlin D Bachi A Wu X. Vesicular stomatitis virus matrix protein inhibits host cell gene expression by targeting the nucleoporin Nup98. Mol Cell (2000) 6(5):1243–52. 10.1016/S1097-2765(00)00120-9 11106761

[43] Ahmed M Brzoza KL Hiltbold EM. Matrix protein mutant of vesicular stomatitis virus stimulates maturation of myeloid dendritic cells. J Virol (2006) 80(5):2194–205. 10.1128/JVI.80.5.2194-2205.2006 PMC1395366 16474127

[44] Gray Z Tabarraei A Moradi A Kalani MR. M51R and Delta-M51 matrix protein of the vesicular stomatitis virus induce apoptosis in colorectal cancer cells. Mol Biol Rep (2019) 46(3):3371–9. 10.1007/s11033-019-04799-3 31006094

[45] Saito T Owen DM Jiang F Marcotrigiano J Gale M Jr. Innate immunity induced by composition-dependent RIG-I recognition of hepatitis C virus RNA. Nature (2008) 454(7203):523–7. 10.1038/nature07106 PMC2856441 18548002

[46] Schnell G Loo YM Marcotrigiano J Gale M Jr. Uridine composition of the poly-U/UC tract of HCV RNA defines non-self recognition by RIG-I. PLoS Pathog (2012) 8(8):e1002839. 10.1371/journal.ppat.1002839 22912574 PMC3410852

[47] Meier E Harmison GG Keene JD Schubert M. Sites of copy choice replication involved in generation of vesicular stomatitis virus defective-interfering particle RNAs. J Virol (1984) 51(2):515–21. 10.1128/JVI.51.2.515-521.1984 PMC254467 6086960

[48] Schubert M Lazzarini RA. Structure and origin of a snapback defective interfering particle RNA of vesicular stomatitis virus. J Virol (1981) 37(2):661–72. 10.1128/JVI.37.2.661-672.1981 PMC171054 6261012

[49] Lazzarini RA Weber GH Johnson LD Stamminger GM. Covalently linked message and anti-message (genomic) RNA from a defective vesicular stomatitis virus particle. J Mol Biol (1975) 97(3):289–307. 10.1016/S0022-2836(75)80042-8 171415

[50] Pfaller CK Mastorakos GM Matchett WE Ma X Samuel CE Cattaneo R. Measles Virus Defective Interfering RNAs Are Generated Frequently and Early in the Absence of C Protein and Can Be Destabilized by Adenosine Deaminase Acting on RNA-1-Like Hypermutations. J Virol (2015) 89(15):7735–47. 10.1128/JVI.01017-15 PMC4505647 25972541

[51] Villarreal LP Breindl M Holland JJ. Determination of molar ratios of vesicular stomatitis virus induced RNA species in BHK21 cells. Biochemistry (1976) 15(8):1663–7. 10.1021/bi00653a012 178352

[52] Plumet S Herschke F Bourhis JM Valentin H Longhi S Gerlier D. Cytosolic 5’-triphosphate ended viral leader transcript of measles virus as activator of the RIG I-mediated interferon response. PLoS One (2007) 2(3):e279. 10.1371/journal.pone.0000279 17356690 PMC1804102

[53] Kurilla MG Piwnica-Worms H Kenne JD. Rapid and transient localization of the leader RNA of vesicular stomatitis virus in the nuclei of infected cells. Proc Natl Acad Sci U S A (1982) 79:5240–4. 10.1073/pnas.79.17.5240 PMC346871 6291035

[54] Yao H Dittmann M Peisley A Hoffmann HH Gilmore RH Schmidt T. ATP-dependent effector-like functions of RIG-I-like receptors. Mol Cell (2015) 58(3):541–8. 10.1016/j.molcel.2015.03.014 PMC4427555 25891073

[55] Killip MJ Young DF Gatherer D Ross CS Short JA Davison AJ. Deep sequencing analysis of defective genomes of parainfluenza virus 5 and their role in interferon induction. J Virol (2013) 87(9):4798–807. 10.1128/JVI.03383-12 PMC3624313 23449801

[56] Killip MJ Young DF Precious BL Goodbourn S Randall RE. Activation of the beta interferon promoter by paramyxoviruses in the absence of virus protein synthesis. J Gen Virol (2012) 93(Pt 2):299–307. 10.1099/vir.0.037531-0 22049094 PMC3352343

[57] Sun Y Jain D Koziol-White CJ Genoyer E Gilbert M Tapia K. Immunostimulatory Defective Viral Genomes from Respiratory Syncytial Virus Promote a Strong Innate Antiviral Response during Infection in Mice and Humans. PLoS Pathog (2015) 11(9):e1005122. 10.1371/journal.ppat.1005122 26336095 PMC4559413

