# Supplementary material for: Defective Interfering Genomes and the Full-Length Viral Genome Trigger RIG-I After Infection With Vesicular Stomatitis Virus in a Replication Dependent Manner
Source: Front Immunol. 2021 Apr 30;12:595390. doi: 10.3389/fimmu.2021.595390 (PMC8119886; doi:10.3389/fimmu.2021.595390)
Supplement: Supplementary file 1 [file DataSheet_1.pdf]

Supplementary figures and tables

Figure S1

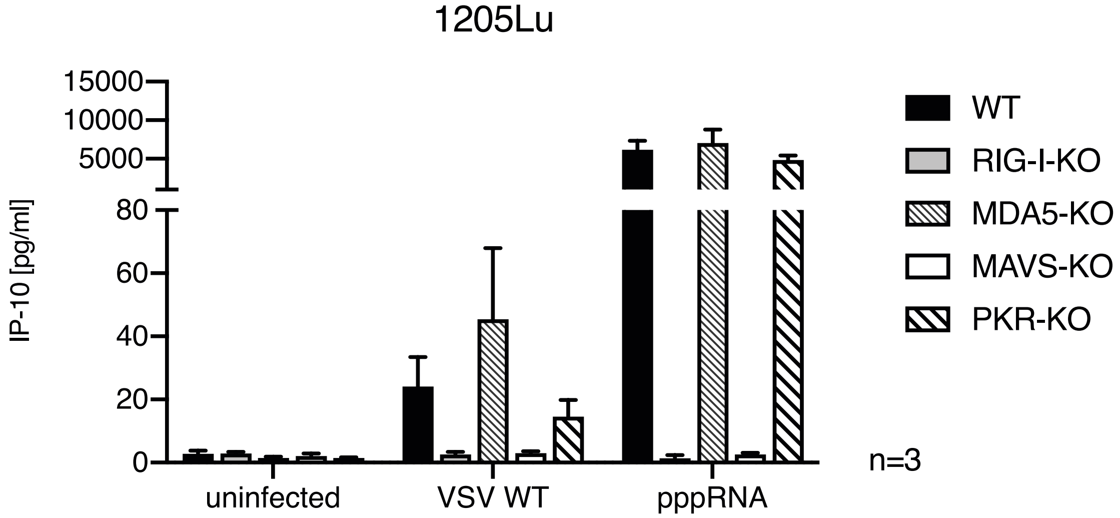

Supplementary figure S1: The early interferon-response after infection with VSV depends on intact RIG-I signaling but does not require MDA5 or PKR.

1 x 10^5 Wt 1205 Lu cells and knockout variants for RIG-I, MDA5, MAVS and PKR generated by CRISPR-Cas9-mediated gene-editing were seeded in triplicates overnight in 96 well plates, and either infected with VSV wt (MOI = 1), lipofected with a known RIG-I stimulating triphosphate RNA (pppRNA; 500 ng/ml) or left untreated. 24 h later IP-10 was measured in the supernatant by ELISA. Data are shown as mean ± SEM of n=3.

Figure S2

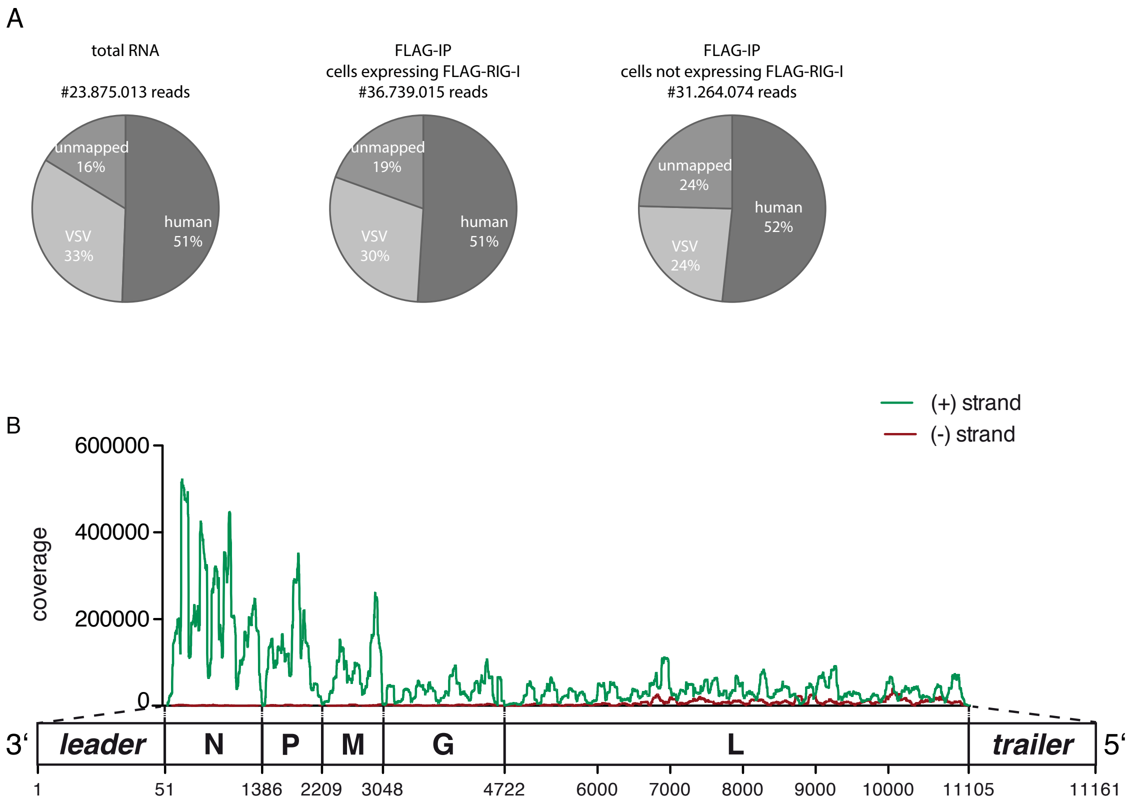

Supplementary figure S2: The majority of RNAs co-immunoprecipitated with anti-FLAG antibody-coated sepharose beads from lysates of VSV-infected cells are unspecifically bound endogenous host RNAs and viral mRNA transcripts.

HEK 293 cells expressing FLAG-RIG-I or not were infected with VSV (MOI=1). 9 hours later cell lysates were prepared and either used directly for RNA isolation or first immunoprecipitated with anti-FLAG antibody-coupled sepharose beads. FLAG-RIG-I/RNA complexes were then eluted from the beads and RNA was isolated from eluates. RNA from the input and RNA from the eluate was used to generate cDNA libraries for next generation sequencing on an Illumina Genome Analyzer. Reads were aligned to the human genome and the VSV reference genome. (A) The relative proportions of reads aligning to the VSV genome, the human genome and unaligned reads were calculated for each condition. One representative experiment of n=2 is shown B) Sequencing reads of a cDNA library generated from VSV-infected cells prior to immunoprecipitation were aligned to the VSV genome reference in positive orientation (green) and negative orientation (red) and are depicted on the y-axis as coverage defined as the number of sequence reads that contain each specific position of the reference genome represented on the x-axis. The x-axis has a resolution of single nucleotide-positions. A schematic representation of the VSV genome underlines the x-axis and is in areas of the trailer and leader sequences not true to scale. One representative experiment out of n=2 is shown.

Figure S3

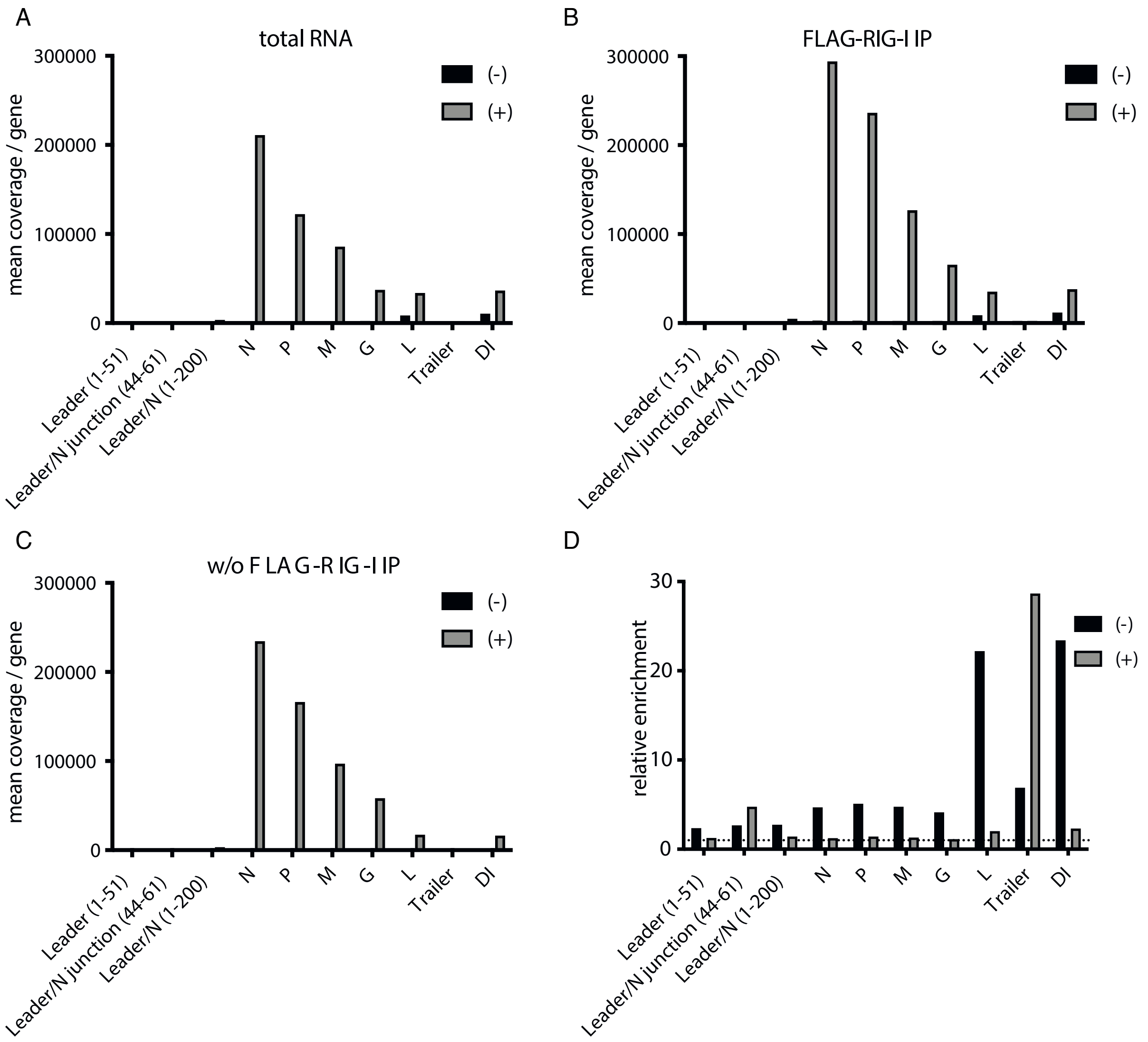

Supplementary figure S3: RIG-I-associated RNAs after VSV infection phenocopy the viral mRNA gradient present in the cytoplasm and are specifically enriched for defective-interfering genomes and genomic VSV sequences in negative orientation.

HEK 293 cells expressing FLAG-RIG-I or not were infected with VSV (MOI=1). 9 hours later cell lysates were prepared and either used directly for RNA isolation (A) or first immunoprecipitated with anti-FLAG antibody-coupled sepharose beads. RNA from the input and RNA from the eluate was used to generate cDNA libraries for next-generation sequencing on an Illumina Genome Analyzer. Sequence reads were aligned with the cRNA sequence of the VSV genome and a de-novo assembled sequence of the 4719 nucleotides long panhandle DI genome identified in figure 5. For the VSV genes N, P, M, G and L, the trailer and leader sequence (nucleotide 1 - 51), the leader-N junction (nucleotide 44 - 61) the leader-N region (nucleotide 1 – 200) as well as the DI genome the mean coverage with reads in positive (+) and negative (–) orientation was calculated by counting the number of all reads aligning to one of the depicted gene sections normalized to the length of the respective gene section. Data are shown from RNA isolated prior to immunoprecipitation (A) or co-immunoprecipitated with anti-FLAG antibodies (B,C) from cells overexpression RIG-I-FLAG (B) or not (C). In D the relative enrichment is depicted by dividing the coverage of B and C. One representative experiment of n=2 is shown.

Figure S4

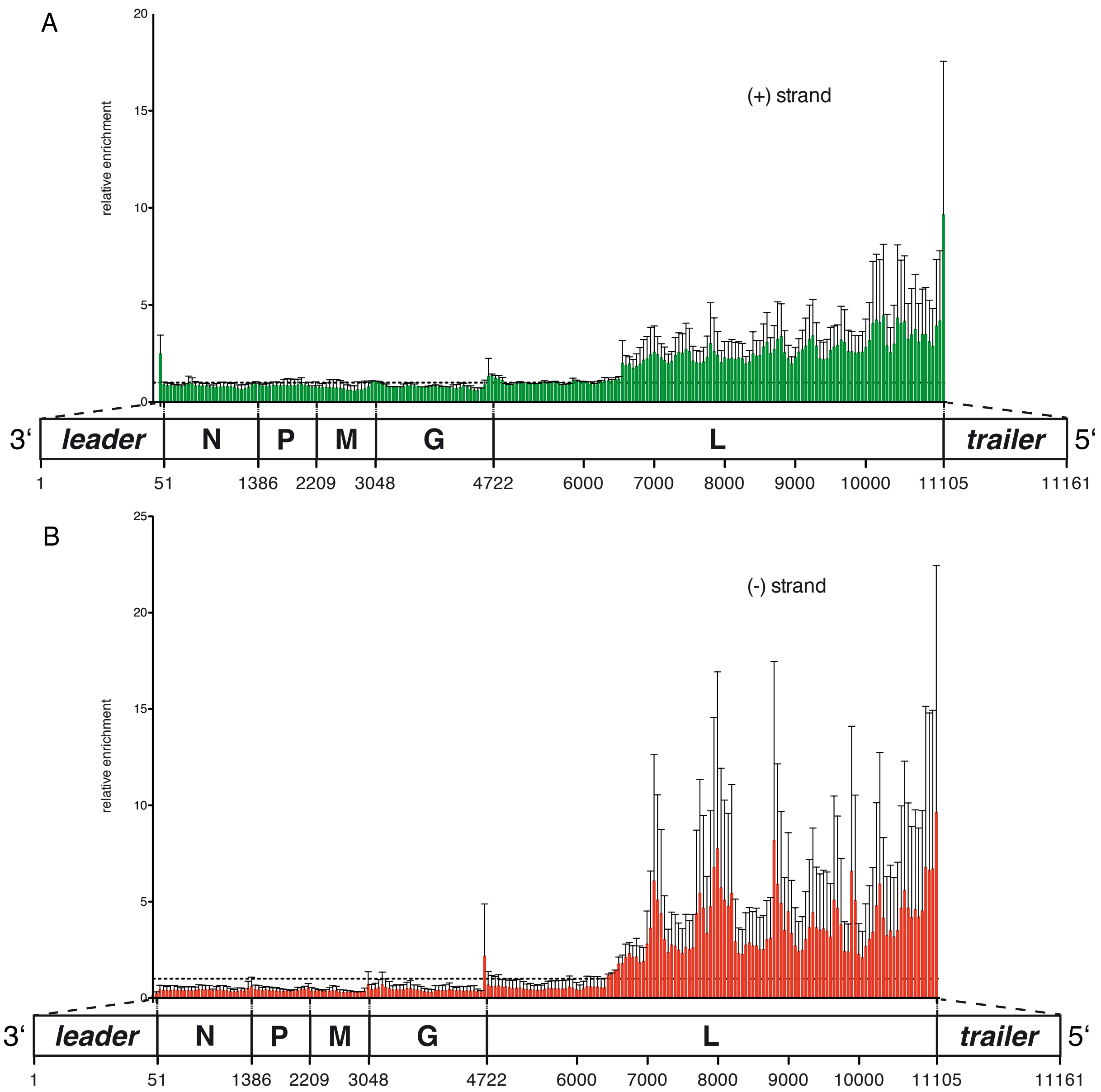

Supplementary figure S4: RIG-I-associated RNAs after VSV infection is enriched for DI genome sequences of positive and negative orientation.

HEK 293 cells (40 x 10^6) expressing FLAG-RIG-I or not were infected with VSV (MOI=1). 9 hours later lysates were prepared and RIG-I/RNA complexes were immunoprecipitated with anti-FLAG-coupled sepharose beads. After elution of the protein/RNA complexes from the beads, RNA was purified from the eluate and used to generate cDNA libraries for next generation sequencing on an Illumina Genome Analyzer. To combine the data from two independent experiments the read sequences were aligned in 50-nucleotide windows to the cRNA sequence of the VSV genome and normalized to the complete number of sequences in each condition aligning to the VSV genome. Data show the relative enrichment on the y-axis by calculating the ratio of the coverage in 50-nucleotide-windows in samples containing RIG-I divided by the coverage in the negative control without RIG-I. A schematic representation of the VSV genome underlines the x-axis and is in areas of the trailer and leader sequences not true to scale. The analysis was performed separately for sequences aligning to the VSV genome in positive orientation (green) (A) and negative orientation (red) (B). The dotted line marks 1. Data are shown as mean +- SD of n=2.

Figure S5

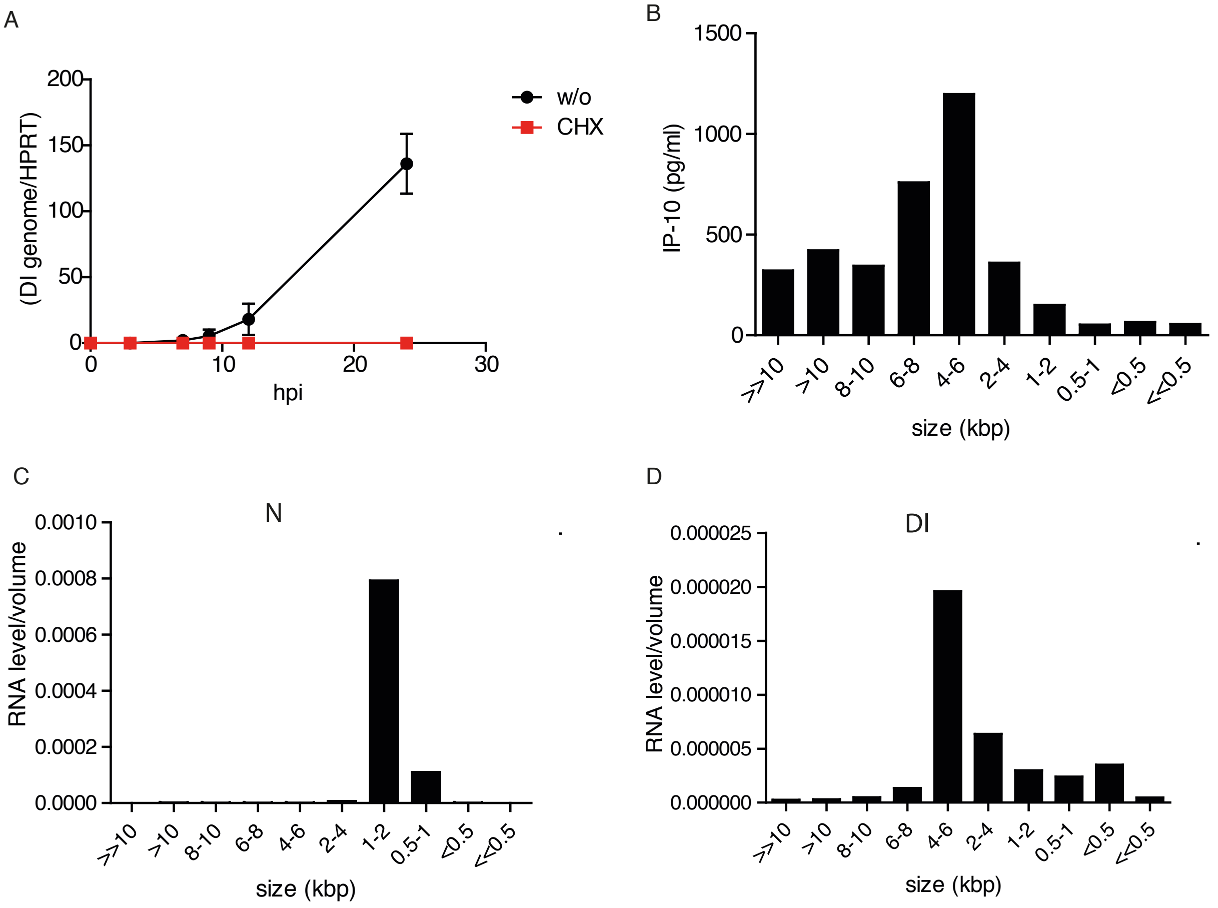

Supplementary figure S5: **After VSV wt infection DI genomes replicate in a cycloheximide-sensitive manner and form the main immunostimulatory RNA that co-immunoprecipitates with RIG-I.**

(A) HEK 293 cells were treated or not with cycloheximide (CHX, 100μg/ml) 30 min prior to infection with VSV (MOI=1) and lysed at the indicated time points post infection. RNA was isolated and analysed by RT-qPCR using specific primers for the 4719 nucleotides DI genome shown in figure 5. Data are shown as mean ± SEM of n=3 independent experiments. (B-D) HEK 293 cells expressing FLAG-RIG-I were infected with VSV (MOI=1). 24 hours later lysates were prepared and RIG-I/RNA complexes were immunoprecipitated with anti-FLAG antibody-coupled sepharose beads. After elution RNA was purified from the eluate and subjected to size-dependent separation on an agarose gel. RNA of the indicated sizes was recovered from ten slices and (B) used for re-transfection into 1205Lu cells. IP-10 in the supernatant was measured 24 h after transfection. (C, D) To validate the size-dependent separation of VSV RNA species equal volume of RNA from each slice was analyzed by RT-qPCR for the presence of the N-RNA (C) and DI genome (D). Quantities were calculated as $2^{(\text{-CP-value})}$. Shown are data of one experiment.

Figure S6

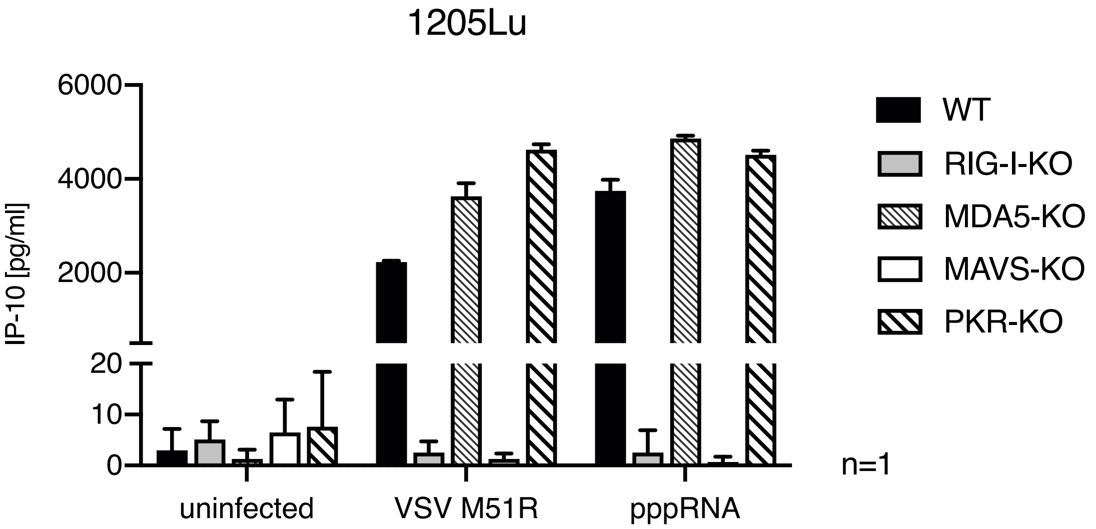

Supplementary figure S6: The early interferon response after infection with VSV M51R depends on intact RIG-I signaling but does not require MDA5 or PKR.

1 x 10^5 Wt 1205 Lu cells and knockout variants for RIG-I, MDA5, MAVS and PKR generated by CRISPR-Cas9-mediated gene-editing were seeded in triplicates overnight in 96-well plates, and either infected with VSV M51R (P0) (MOI = 1), lipofected with a known RIG-I stimulating triphosphate RNA (pppRNA;500 ng/ml) or left untreated. 24 h later IP-10 was measured in the supernatant by ELISA. Data are shown as mean ± SEM of triplicates (n=1) and are representative for three closely related experiments with similar results.

Figure S7

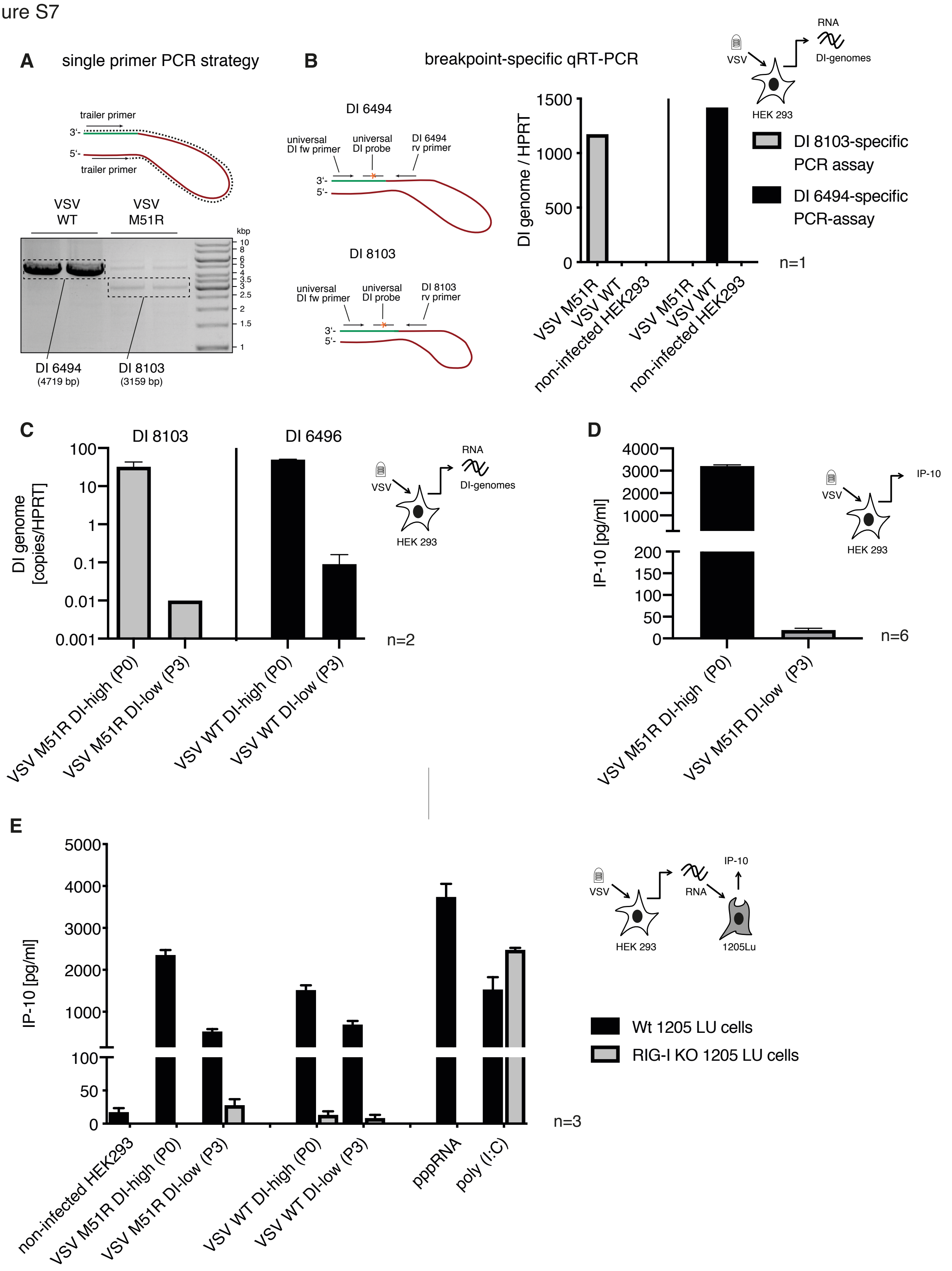

Supplementary figure S7: Stocks of the VSV M51R mutant containing a 3159 nucleotide long defective-interfering genome lose most of their RIG-I stimulating capacity after depletion of the DI genome

(A) RNA isolated from BHK cells 24 h after infection with either VSV wt stocks or stocks of the VSV M51R mutant was reverse transcribed and amplified using the single primer PCR-

strategy schematically depicted. This approach is based on a single primer that binds RNA-sequences contained in the trailer sequence of VSV and only gives a product in the presence of sequences that contain the trailer sequence on the 5' end and its reverse complementary sequence on the 3' end. PCR products were analyzed on an agarose gel and the known 4719 bp long DI genome with its breakpoint at position 6494 (DI 6494) in the VSV wt stocks served as a positive control for the assay. (B) Reisolation of DNA and sequencing of the 3159 bp band from the VSV M51R stock identified a copyback DI genome with a breakpoint at position 8103 (DI 8103) and allowed the design of specific RT-qPCR assays for the two DI genomes schematically depicted. Analysis of RNA isolated from HEK cells infected for 24 h (MOI = 1) with VSV M51R or VSV wt gave specific signals in these RT-qPCR assays without "cross contamination". RNA isolated from non-infected HEK cells served as a negative control.

(C-E) HEK 293 cells were infected with VSV M51R or VSV wt (MOI =1) using virus stocks containing either high (P0) or low (P3) amounts of DI genomes. 24 h post infection supernatants were harvested, the infected cells were lysed and their RNA was isolated. (C) The isolated RNA was transcribed to cDNA and was analysed by RT-qPCR for the amount of DI genomes using the indicated DI-specific PCR assays. (D) IP-10 was measured in the harvested supernatants by ELISA. (E) The isolated RNA was retransfected into 1205Lu wt and RIG-I deficient cells (100 ng/100 μl) using RNAiMax lipofectamin. IP-10 in the supernatant was measured 24 h after transfection by ELISA. pppRNA (500 ng/ml) and Poly (I:C) (100ng/ml) were used as RIG-I-dependent and RIG-I-independent controls respectively. Data are represented as mean ± SEM of n=1-6 independent experiments as indicated.

Figure S8

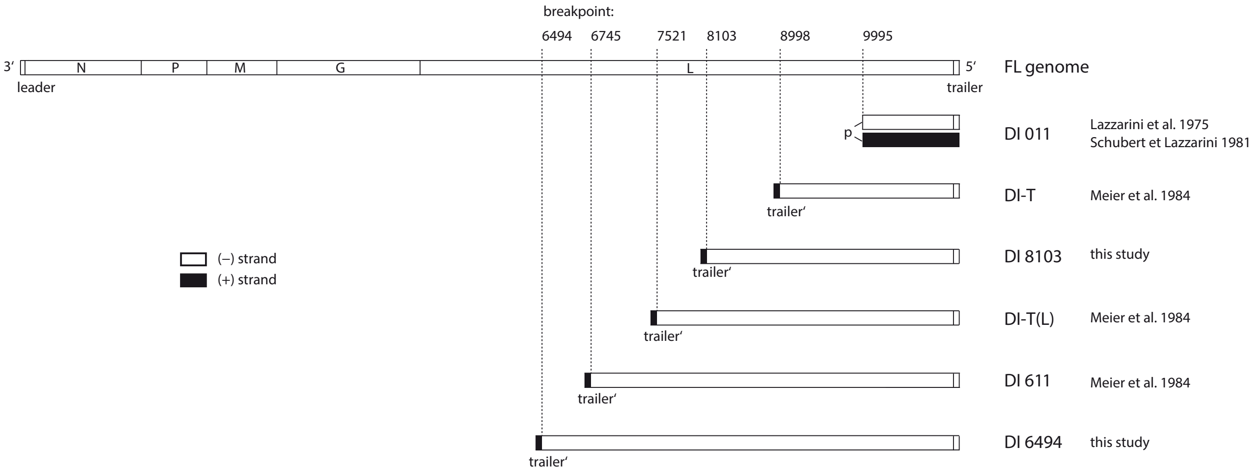

Supplementary figure S8: Graphical representation and comparison of published examples for defective interfering genomes in VSV

Several VSV defective interfering (DI-) genomes with distinct breakpoints within the L gene have been described so far (Lazzarini et al., 1975; Schubert and Lazzarini, 1981; Meier et al., 1984). These DI genomes all consist of the 5'-sequence of the full-length (FL) genome that extends to the L gene to varying degrees. The copy-back DI genomes have a reverse complementary trailer sequence (*trailer'*) attached to their 3'-end.
Figure adopted and modified from (Meier et al., 1984)

Lazzarini, R.A., Weber, G.H., Johnson, L.D., and Stamminger, G.M. (1975). Covalently linked message and anti-message (genomic) RNA from a defective vesicular stomatitis virus particle. *J Mol Biol* 97(3), 289-307.

Meier, E., Harmison, G.G., Keene, J.D., and Schubert, M. (1984). Sites of copy choice replication involved in generation of vesicular stomatitis virus defective-interfering particle RNAs. *J Virol* 51(2), 515-521.

Schubert, M., and Lazzarini, R.A. (1981). Structure and origin of a snapback defective interfering particle RNA of vesicular stomatitis virus. *J Virol* 37(2), 661-672. doi: 10.1128/JVI.37.2.661-672.1981.

Supplementary table 1: RT-qPCR primer list

Name	Probe	Sequence Primer 1 (left) (5'-3')	Sequence Primer 2 (right) (5'-3')	Region on VSV genome covered by amplificate (3'-5')	Reverse Transcription Primer
Human HPRT	Roche UPL #73	TGACCTTGATTTATTTTGCATA CC	CGAGCAAGACGTTCAGTCCT	-	Random Hexamer Primer (Thermo scientific)
Leader	Custom made (TIB MOLBIOL) 6FAM-CCTGAGCCTTTTAATG ATAA--BBQ	ACGAAGACAAACAAACCAT	GCCTCTCATGCTGACGAAT	1 - 50	Custom made (TIB MOLBIOL) GCCTCTCA TGCTGACGAATTT TGAGAGGC AAAGTTTC A
Leader/N	Roche UPL #2	CGAAGACAAACAAACCATTATT ATCA	GTTGTCAATGATTCTCTTGACTG TAAC	1 – 96	Random Hexamer Primer
N	Roche UPL #85	CGAAGACAAACAAACCATTATT ATCA	TCTGCAACTTCTCGGTTCAA	766 - 830	Random Hexamer Primer
P	Roche UPL #7	GCAGAGTGCACATTTGAAGC	AGTTATCTGGCGCTCCTTCAT	1858 - 1917	Random Hexamer Primer
M	Roche UPL #60	GCGAAGGCAGGGCTTATT	GCTCTGGTACATTGAGCATGG	2653 - 2718	Random Hexamer Primer
G	Roche UPL #71	TGGTTCGAGATGGCTGATAA	ACTTGACCCTTCTGGGCATT	3831 - 3899	Random Hexamer Primer
L	Roche UPL #62	CCTTTAGAAGGGAATTGGAAG AA	TCTGCCGACTTGATAGGATTG	8765 - 8824	Random Hexamer Primer
L/Trailer	Custom made (TIB MOLBIOL) 6FAM-TCTTGTGGTTTTTATT TTTTATCTGG--BBQ	CATGAGGAGACTCCAAAC	GACGAAGACCACAAAACC	11068 - 11161	Random Hexamer Primer

Trailer	Custom made (TIB MOLBIOL) 6FAM-TCTTGTGGTTTTTATTTTTTATCTGG--BBQ	GGCTTTGATCCTTAAGACC	GACGAAGACCACAAAACC	11103 - 11161	Random Hexamer Primer
IFN-β	Roche UPL #25	CGACACTGTTCGTGTTGTCA	GAGGCACAACAGGAGAGCAA	-	Random Hexamer Primer
DI6494	Custom made (TIB MOLBIOL) 6FAM-TCTTGTGGTTTTTATTTTTTATCTGG--BBQ	CGCGGGACGAAGACCACAAAA	GCCGTTTGATAACTTCCTTTGGG		Random Hexamer Primer
DI8103	Custom made (TIB MOLBIOL) 6FAM-TCTTGTGGTTTTTATTTTTTATCTGG--BBQ	CGCGGGACGAAGACCACAAAA	CGTGAACTAAAGACGTCATGGATC		Random Hexamer Primer
